# Combined Mouse Retinal Optoretinography/Electroretinography System to Study Light-Evoked Responses in Animal Models of Retinal Degeneration

**DOI:** 10.1167/iovs.67.1.39

**Published:** 2026-01-20

**Authors:** Hang Chan Jo, Ewelina A. Pijewska, Ratheesh K. Meleppat, Ravi S. Jonnal, Ala Moshiri, Dae Yu Kim, Robert J. Zawadzki

**Affiliations:** 1 Department of Electrical and Computer Engineering, College of Engineering, Inha University, Incheon, Republic of Korea; 2 UC Davis EyePod Small Animal Ocular Imaging Laboratory, Department of Cell Biology and Human Anatomy, University of California, Davis, Davis, California, United States; 3 Center for Human Ophthalmic Imaging Research (CHOIR), UC Davis Eye Center, Department of Ophthalmology and Vision Science, University of California, Davis, Sacramento, California, United States; 4 Department of Ophthalmology and Vision Science, University of California, Davis, Sacramento, California, United States; 5 Institute of Physics, Faculty of Physics, Astronomy and Informatics, Nicolaus Copernicus University, Toruń, Poland

**Keywords:** optoretinography (ORG), electroretinography (ERG), optical coherence tomography (OCT), light-evoked retinal response, animal models

## Abstract

**Purpose:**

To study the correlation between optoretinogram (ORG) and the underlying phototransduction-initiated physiology, we irradiated mouse retinas with visible light centered at a 482-nm wavelength to induce bleach-initiated morphologic changes in the outer retinal layers, including photoreceptors and the retinal pigment epithelium (RPE).

**Methods:**

A 482-nm light-emitting diode was used for short-pulse irradiation of the retina over a large field of view in the three groups of mice, including disease models, while acquiring optical coherence tomography (OCT) image sequences using a custom-built OCT system combined with a commercial electroretinogram (ERG) system. The visible light exposure was adjusted to vary the total light energy (number of photons) delivered to the retina, enabling observation of the bleach-level dependence of the ORG and ERG signals.

**Results:**

Light-driven thickness increments in the outer retinal layers, including the photoreceptors and RPE, were observed in wild-type (WT) albino and pigmented mice. However, the energy of light stimuli did not produce a response in the retina of the rd10 mouse model. The lack of a full-field ERG response in the same animals also confirmed this observation. These suggested that phototransduction in the 3-month-old rd10 mice could not be initiated.

**Conclusions:**

The ORG and ERG measurements recorded under various light stimuli reveal the retina's neural function. The ORG and ERG signals from the WT albino and pigmented mice exhibited thickness increments in the ORG and an increase in amplitude of a-waves and b-waves in the ERG, which were linked to phototransduction; however, these signal trends were not observed in the rd10 mice. Therefore, the ORG/ERG system could be an attractive instrument that provides both localized structural and global functional information about the investigated retina, allowing for detailed studies of neural function suppression in animal models of retinal degeneration.

Recently, numerous optical coherence tomography (OCT) studies have focused on observing and modeling light-evoked changes in retinal layers to probe the neural function of the retina during phototransduction.^1^^–^^9^ These studies have referred to the analysis of visible light-evoked responses in retinal layers as the optoretinogram (ORG).^7^^,^^10^^,^^11^ When photons are captured by opsins in the outer segment of photoreceptors, the retinal neurons undergo various biochemical and biophysical changes, including charge-dependent alterations in disc–membrane forces and osmotic pressure, which cause water movements and, consequently, nanometer-scale changes in retinal layer thickness.^12^ These responses generate specific effects, including the deformation of the length of the outer segments (OSs) of photoreceptors.^7^^,^^13^ The clinical ORG mainly measures light-driven morphologic changes, such as the shrinkage and elongation, of the OS lengths of the cones. Clinical and preclinical ORG studies have reported using retinal images acquired using various OCT systems, which could be categorized in terms of the OCT image acquisition method, such as full-field OCT,^3^^,^^14^^,^^15^ line-scan OCT,^4^ and raster scan OCT,^2^^,^^8^^,^^16^ as well as the method of ORG processing, such as intensity-based,^6^^,^^16^ phase-based distance,^2^^,^^8^ and phase-based velocity ORG.^5^ Although ORG signals caused by light stimulation in dark-adapted retinas were observed in these studies, correlations between these effects, the physiology underlying processes, and their connections with phototransduction have been modeled in just a few instances.^16^^–^^18^ Hence, simultaneous measurements of ORG responses with electroretinogram (ERG) should help link known phototransduction steps with the kinetics of the ORG signal. ERG has been widely accepted as an objective method for probing retinal function by measuring the electrical responses of retinal neural activity after a light stimulus.^19^^,^^20^ It detects the hyperpolarization and depolarization of several retinal cells, including photoreceptors, bipolar cells, and Müller glial cells, during phototransduction and transmission of the electrical activity toward the brain. To measure the global electrical response of the entire retina, known as the full-field ERG, the retina surface should be illuminated with Ganzfeld illumination to ensure uniform irradiation. However, instead of using this illumination method, our previous OCT/ORG studies relied on flying spot scanning patterns for visible light stimulation.^2^^,^^16^ These irradiation patterns resulted in time differences in spatial light stimulation due to time-dependent scanning. In addition, since the light source for stimulation continuously irradiated the retina during a single OCT volume scan (∼1 second), the previous light-stimulating method was inadequate for short flash stimulation across a large field of view, which is required for generating a full-field ERG signal.

In this study, we developed and built a custom mouse OCT/ORG system with an independent Ganzfeld visible light illumination channel. This system was combined with a commercial ERG detection subsystem, enabling the simultaneous investigation of full-field ERG and ORG signals. Using OCT BM-scan (serial cross-sectional B-scans acquired at the same position) sequences of the mouse retina, ORG signals were obtained through phase-based quantification of the light-evoked morphologic changes in the outer retina. We simultaneously acquired several ORG and ERG signals using different stimulus energies in various mouse models, including wild-type (WT) albino and pigmented mice, as well as the rd10 retinal degeneration model.

## Methods

### Development of Combined ORG and Full-Field ERG Systems

**Figure 1.**
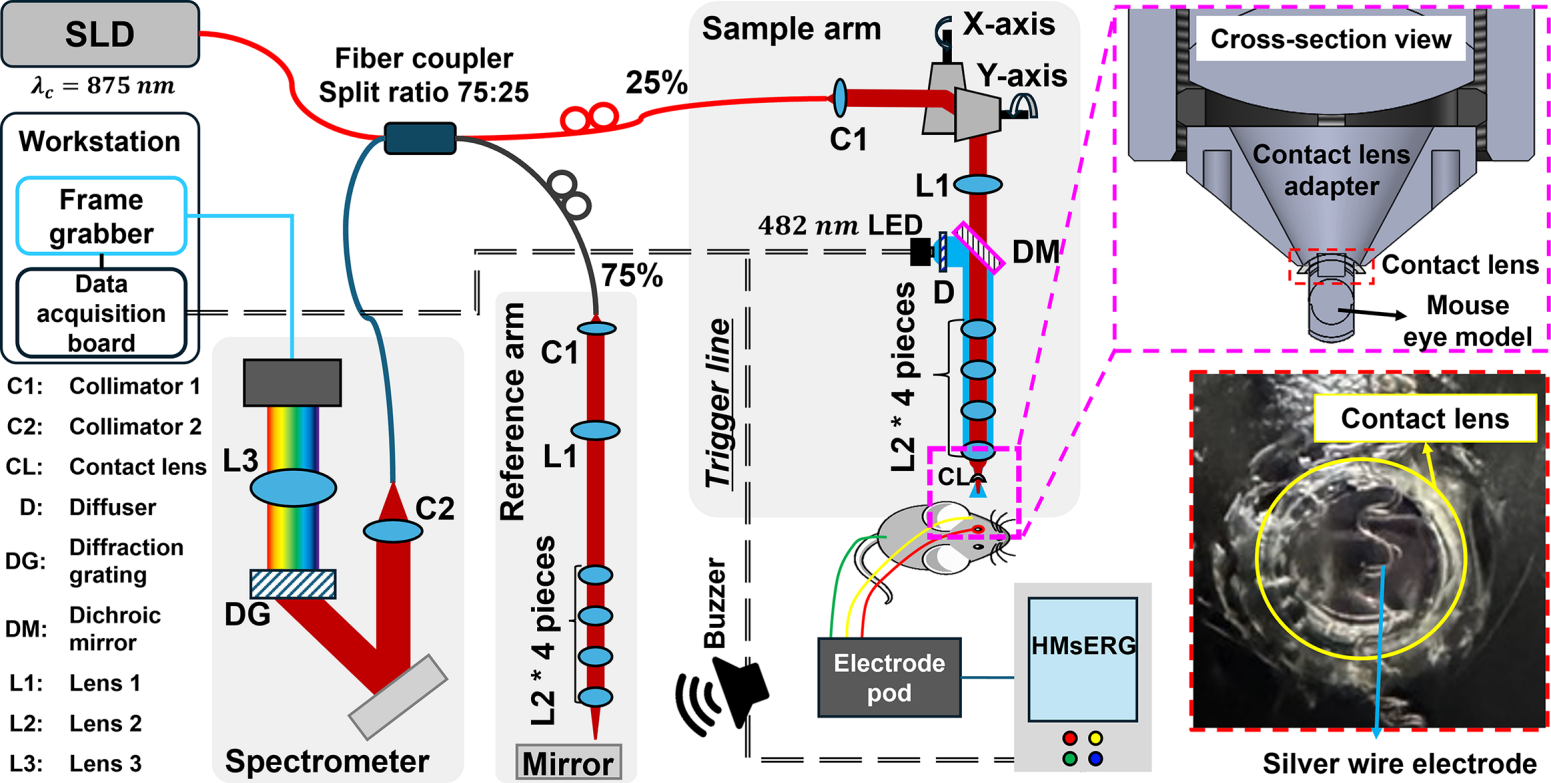
Schematic of the custom-built OCT/ORG system combined with a commercial ERG system (HMsERG) to observe the visible light stimulation–driven scotopic retinal response in the living mice. The data acquisition board in the workstation (PCIe-6363; National Instruments, Austin, TX, USA) controlled the OCT operation, visible light LED channel, and ERG measurements through the trigger signal, allowing for the simultaneous measurement of ORG (OCT) and full-field ERG signals. Additionally, a buzzer shared the trigger line, indicating the timing of the light stimulation and ERG measurement in a dark room. The silver wire electrode of the ERG system was located between the contact lens at the end of the imaging probe and the mouse cornea. A photograph of the developed ORG + ERG system is shown in Supplementary Figure S2.

For simultaneous acquisition of ORG and ERG signals, we constructed a custom OCT system that shared light stimulation for ORG, ERG, and triggered data acquisition by ERG electrodes. The OCT system provided a lateral resolution of ∼4.9 µm in the mouse retina and a theoretical axial resolution of ∼1.9 µm in the air from the central wavelength (875 nm) and bandwidth (150 nm) of a broadband superluminescent diode (SLD) light source (MT-870-HP; Superlum, Carrigtohill, Ireland). The corrected axial resolution was ∼1.4 µm, considering the average refractive index of the mouse retina (1.35).^21^ The output beam power of the SLD was 6.9 mW, which was split by a 75:25 fiber coupler (TW850R3A2; Thorlabs, Newton, NJ, USA) to deliver 825 µW at the mouse cornea. The sample arm included an optical stimulation path using an achromatic doublet, a mouse contact lens, and the visible light (482 nm) light-emitting diode (LED) source (M490L4; Thorlabs). A pair of galvo scanners (6215H; Cambridge Technology, Bedford, MA, USA) enabled x- and y-axis scanning with 100-kHz A-scan rates. A line scan camera (spL4096-140km; Basler AG, Ahrensburg, Germany) of a custom-built spectrometer provided 100-kHz acquisition rates for a spectrum image corresponding to an A-scan. The scanners allowed scanning for the OCT volume and en face images with a field of view (FOV) of 50° × 50° corresponding to 1.7 × 1.7 mm of the mouse retina. While the spectrometer collected OCT spectral fringe data during animal experiments with light stimulation, a commercial ERG system (HMsERG; Xenotec, d/b/a OcuScience, Henderson, NV, USA) simultaneously recorded ERG signals. The scanning patterns and LED light bleaching protocols for the experiments are detailed in the “Protocol for Data Acquisition and Light Stimulation” section. To acquire ERG signals, a silver wire electrode was submerged in a gel (GenTeal Tears; Alcon, Fort Worth, TX, USA) between the contact lens and the mouse cornea. This gel filled the gap in the contact region, preventing the eye from drying and maintaining ocular transparency. An experimental schematic and photograph of the contact region with the silver wire electrode are shown in Figure 1.

**Figure 2.**
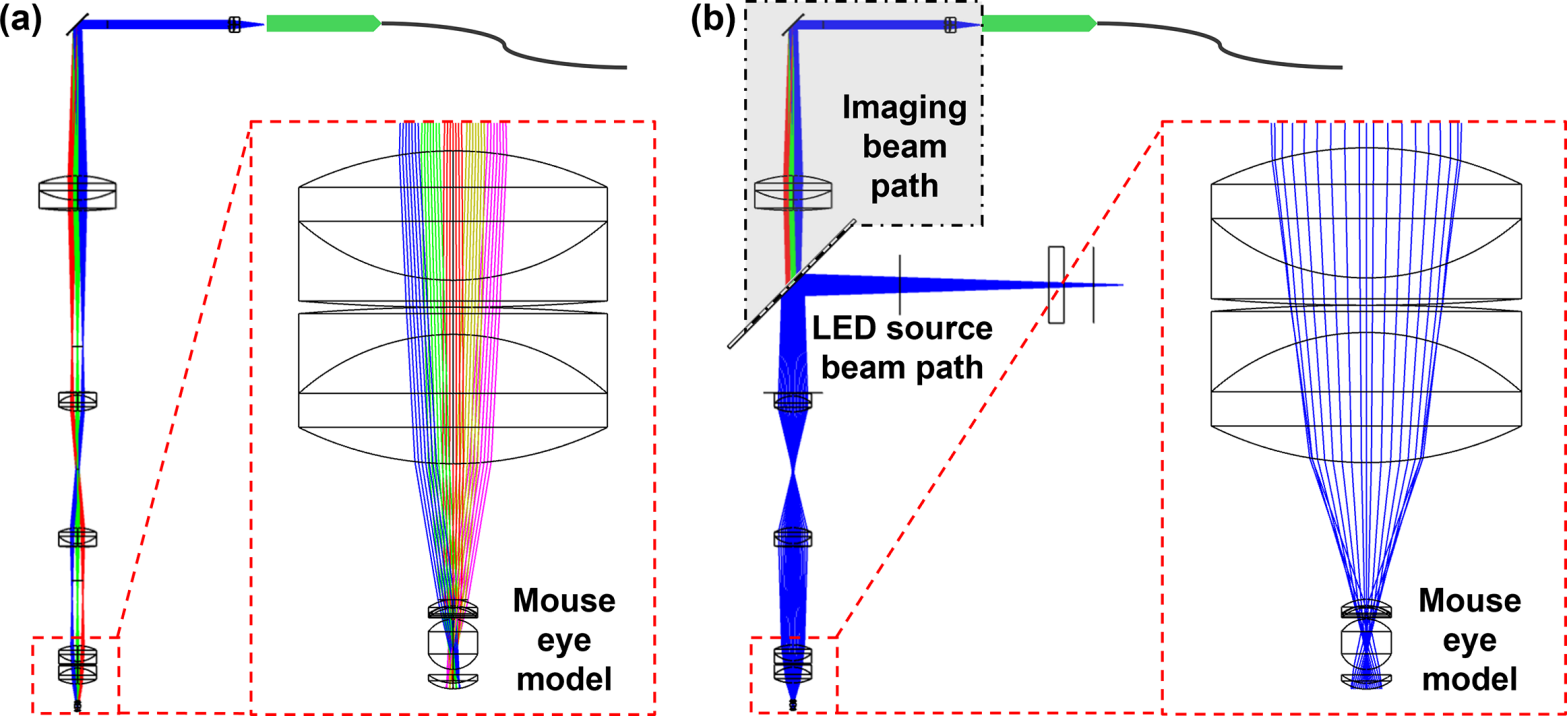
Optical design of the sample arm for mouse retinal imaging and stimulus illumination. (**a**) Optical simulation of one-axis scanning with the mouse eye model. Each color represents a beam path for a different scanning angle. The mouse model has been used in previous studies.^22^^,^^23^ (**b**) LED full-field light illumination through the common beam path to the mouse eye, modeled with a reflection by a dichroic mirror.

The sample arm, including the imaging probe and visible light irradiation channel, was developed through optical design and three-dimensional computer-aided mechanical design using OpticStudio (Ansys, Inc. Canonsburg, PA, USA) and SolidWorks (Dassault Systems, Velizy-Villacoublay, Yvelines, France), respectively (Fig. 2a). A mouse eye model described in a previous study was used as a sample in the optical simulation involving beam scanning.^22^ Figure 2b shows the common beam path in the sample arm and the focal point of the LED beams in the mouse eye model. All optical elements from the optical simulation were exported as a computer-aided design file and mounted on optomechanical models. A simple beam profiling method utilizing a complementary metal oxide semiconductor sensor was used to measure the FOV illuminating the retina. A photograph of the developed ORG + ERG system is shown in Supplementary Figure S2, and the details of the beam profiling method are described in Supplementary Figure S3.

### Calculation of Rhodopsin Bleach Ratio Equivalent Stimulus Light Energy

We calculated the energy required for full-field light illumination based on a previous study to bleach different ratios of rhodopsin in mice. The aforementioned study reported the absolute photosensitivity of rhodopsin (*P_a_*) in situ for light measured at the cornea to be 7.0 × 10^−9^ µm^2^ at the mid-wavelength of 501 nm.^24^ Accordingly, the reciprocal of this *P_a_* represents the light energy (1.43 × 10^8^ photons/µm^2^ ≈ (7.0 × 10^−9^ µm^2^)^−1^) required to bleach 100% of the rhodopsin. Applying this value to 482-nm wavelength light, the total flash energy of the stimulating beam should be 0.059 nJ/µm^2^ for 100% bleaching. In these experiments, mouse eyes were exposed to light stimulation energy corresponding to 10%, 20%, and 40% bleaching levels. The field illumination area on the retina surface was estimated to be 4.061 mm^2^ using the formula for the side surface area of a solid of revolution, and the detailed estimation is described in Supplementary Figure S4. Then, the LED module was operated with 1.843 mW (80% of the maximum output power of the light source) for 13 ms (10% bleaching), 26 ms (20% bleaching), and 52 ms (40% bleaching), using the calculations for the light-exposed area, which are presented in the section 4 of the Supplementary Document.

### Phase Difference Stability Measurements

The analysis of retinal thickness variation using phase-based position estimation required a stable phase during the whole OCT data acquisition. The measured phase differences between two positions from the same A-scan (z-axis) of each consecutive BM-scan series (signal-to-noise ratio [SNR] = 58.54 dB without final lens corresponding to mouse cornea and lens) showed phase stability in radians or nanometers for phase average and its standard deviation (SD) values. The phase differences between two axial positions over 500 sequential BM-scans exhibited a mean (SD) of 0.01 (0.13) rad. These measurement error values were compensated for the actual mouse retina imaging based on the SNR value (29.98 dB) of the retinal imaging. Converting these values to optical path length (OPL) nanometers resulted in a mean error of 0.72 nm (0.014 rad) and a standard deviation of 9.464 nm (0.182 rad). The phase difference stability measurement and results are explained in Supplementary Figure S5 and section 5 of the Supplementary Document.

### In Vivo Mouse Experiments

All mouse husbandry and handling were in accordance with protocols approved by the University of California Animal Care and Use Committee, which strictly adheres to all National Institutes of Health guidelines and satisfies the ARVO guidelines for animal use. Three mouse strains with two females in each group were used to test performance of the ERG/ORG system: WT albino (B6(Cg)-Tyrc-2J/J, two females, 6 weeks of age), WT pigmented (C57BL/6, two females, 3 months of age), and retinal degeneration 10 (rd10, two females, 3 months of age) on the pigmented background (C57BL/6) line. WT albino was used to validate the performance of the developed OCT system for acquiring ORG signals by measuring the distance between two sharply defined layers. Time course measurement of the length between the external limiting membrane (ELM) and Bruch's membrane (BrM) could provide outer segment deformations induced by light stimulation. Although the ELM and BrM were well defined in albino mice, in the case of pigmented mice, melanin granules (size 0.6∼1.0 µm) could lead to Mie scattering in the retinal pigment epithelium (RPE) layer, represented as a hyperreflective region, which disturbed BrM imaging in the retina image.^25^^,^^26^ Comparison of B-scans between albino and pigmented mice in Supplementary Figure S6 shows the capability for imaging BrM in albino mice. Based on the validated performance of the OCT system, the RPE band in pigmented mice was also used as one of the layers for ORG acquisition. Unlike the albino mice group, the age of WT pigmented and rd10 mice was selected as 3 months to show distinct differences between the normal and severe disease groups (photoreceptors completely reduced).^27^^,^^28^ According to the previous study, 70-day-old rd10 mice had severe retinal degeneration, including a complete reduction of outer segments of cone and rod cells.^27^^,^^28^ Supplementary Figure S7 presents the B-scan image of 3-month-old rd10 mice, showing a thinned and degenerated retinal layer at 67 µm (normal, 137.5 µm), corresponding to the OPL to RPE layers. Only the retina nerve fiber layer/ganglion cell layer, inner plexiform layer, inner nuclear layer, and RPE remained detectable in 3-month-old rd10 mice, which was also reported by Pang et al.^29^ We believe the age and OCT B-scan images were sufficient evidence to suggest that the rd10 model's retina underwent thorough degeneration. For the ORG experiments, all mice were dark-adapted for 2 hours inside a fully covered dark box. A gas anesthesia system (V-10 Mobile; VetEquip, Livermore, CA, USA) supplied an O_2_ mixture with isoflurane gas in various concentrations. A 4% concentration was used for the initial step in the anesthesia induction chamber before placing the mice in the bite bar for inhalation anesthetic delivery on the animal stage. A 2% isoflurane concentration was subsequently supplied during the imaging session. While the mice were undergoing imaging, the isoflurane gas content was maintained within a 1.7% to 2.5% range to stabilize anesthesia and breathing. Before the imaging experiments, mouse eyes were dilated using eye drops (tropicamide and phenylephrine). A custom heating pad and disposable handwarmer heated the platform for the mouse stage and the bite bar to maintain the temperature while the mice were anesthetized.

### ERG Subsystem

The ERG device (HMsERG) measured the light-evoked electrical response during the hyperpolarization and depolarization of the retinal cells. The ERG electrodes comprised a needle-type ground, a reference electrode, and a corneal surface electrode made of silver wires. To measure the ERG signals, the ground electrode was placed near the tail, far from the eye, and the reference electrode was placed on the cheek under the eye. The silver wire was interleaved between the contact lens of the imaging probe and the cornea. The ERG system was customized by the manufacturer to perform a slave role in the trigger function to synchronize ERG acquisition, light stimulation, and OCT imaging for ORG signals. After data acquisition, a signal processing code developed in the LabVIEW software filtered the raw electrical signals for the ERG using a 60-Hz notch, a 0.05- to 1000-Hz bandpass, and Gaussian filters to reduce noise.^30^ The ERG signals before and after the filtering process are shown in Supplementary Figure S8.

### Protocol for Data Acquisition and Light Stimulation

The experimental protocol was divided into two steps: (1) preparation for BM-scan acquisition (multiple B-scans on the same position) and (2) data acquisition during light stimulation. All experiments, performed in a dark room, are summarized below:

Step 1. Preparation for BM-scan acquisition
  1-1. Dark adaptation of the mouse (2 hours)
  1-2. Initial anesthesia in the induction chamber
  1-3. Placing the mouse on the three-axis stage for imaging and adjusting the concentration of isoflurane gas
  1-4. Dilating mouse pupils with drops (tropicamide and phenylephrine)
  1-5. Dispensing the gel (GenTeal Tears; Alcon) on the cornea and contact lens
  1-6. Inserting needle-type electrodes into the mouse and placing the silver wire electrodes in front of the contact lens
  1-7. Positioning the three-axis stage to attach the mouse cornea to the imaging probe
  1-8. Volume imaging for the en face view (FOV: 1.7 × 1.7 mm) to display the imaging regions on the retina
  1-9. Optimizing the position of the mouse stage for the imaging system to achieve the desired retinal eccentricity
  1-10. Selecting the area for BM-scans

Step 2. ORG and full-field ERG signal acquisition during light stimulation
  2-1. Acquiring time-series baseline data, both OCT and ERG, for 10 seconds without visible light irradiation
  2-2. Acquiring OCT and ERG time series for 10 seconds after visible light stimulation (allowing simultaneous ORG and ERG signal acquisition)

**Figure 3.**
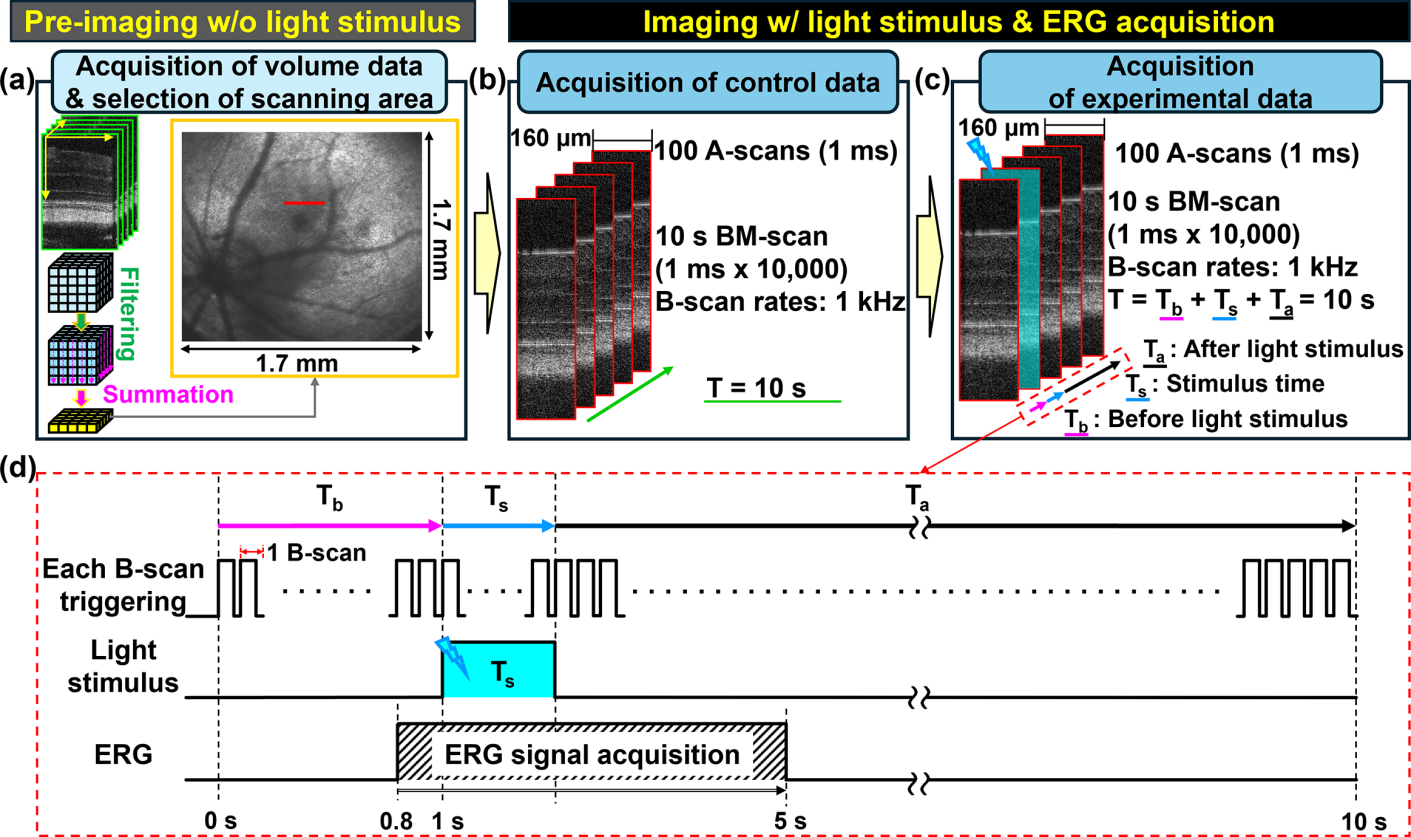
Protocols for acquiring OCT volume data for en face projection views and BM-scans. The volume data were acquired to determine the location of the B-scan area through the live en-face view. A series of B-scans was required to analyze the temporal evolution of ORG signals. (**a**) Imaging for the selection of the scanning area before the main experiments. Selection of a scanning area for BM-scan acquisition using a reconstructed en face projection image of the retina during imaging sessions via fast Fourier transform (FFT) intensity data processing. The volume data comprises 1000 × 1000 A-scans, including flybacks of the scanner raster patterns. After selecting the scanning area, the volume data were saved (steps 1-8 through 1-10 in the summarized protocol). (**b**) Baseline data acquisition in a selected scanning range without a visible light stimulus. The baseline data consist of BM-scans acquired for 10 seconds at a 1-kHz B-scan rate. (**c**) The same data acquisition process was repeated along with full-field ERG signal measurement and light stimulation for 10 seconds, including the time before light stimulation (Tb), stimulation time (Ts), and time after stimulation (Ta). ERG signal measurement was restricted to 5 seconds due to the memory limit of the ERG instrument. If the stimulus time, Ts, was varied to adjust light energy, Ta was adjusted to maintain a total elapsed time of 10 seconds. (**d**) Timing diagram depicting imaging, light stimulus, and ERG measurement. ERG measurement starts at 800 ms, which allows better utilization of the limited acquisition time of the ERG subsystem.

The imaging system could display real-time en face OCT projection views via high-pass filtering and summation of the volume data during volume scanning.^31^ Although our previous system utilized a scanning light ophthalmoscope (SLO) for live retinal fundus views during animal positioning, our updated OCT system enabled us to observe retinal en face views without the need for a fundus camera or SLO.^32^ Figure 3a illustrates the simple OCT en face projection view, which yielded the retinal images used in steps 1-8, 1-9, and 1-10 in the experimental protocol. After acquiring BM-scan images without visible light exposure, the digital trigger signals, including BM-scans and ERG signals with light stimulation, were utilized to obtain experimental data. The LED channel provided visible light for a few milliseconds, starting at 1 second after the start of data acquisition. The stimulus flash exposure time (Ts) shown in Figure 3c was adjusted to meet the light energy requirements for each rhodopsin bleach level. The timeline of the digital triggers is illustrated in Figure 3d.

### Postprocessing of OCT Data to Extract ORG Signals

The OCT data processing for extracting ORG signals involved two steps: processing sequential OCT B-scan data and calculating the time-dependent distance between specific retinal layers (ELM to BrM or ELM to RPE). First, custom postprocessing software based on previous studies was used to perform a fast Fourier transform on the dispersion-compensated spectral fringe data after k-linearization and phase calibration.^33^^,^^34^ The OCT image processing provided amplitude and phase information for each pixel in the OCT B-scans, derived from the spectral fringe data acquired by the spectrometer. The phase-based ORG processing software reported in our previous study was employed to calculate the distance between two retinal layers, including OSs. The detailed procedure for ORG processing is explained in Supplementary Figure S9.

## Results

### Light-Evoked ORG and Full-Field ERG Responses in WT Albino Mice

**Figure 4.**
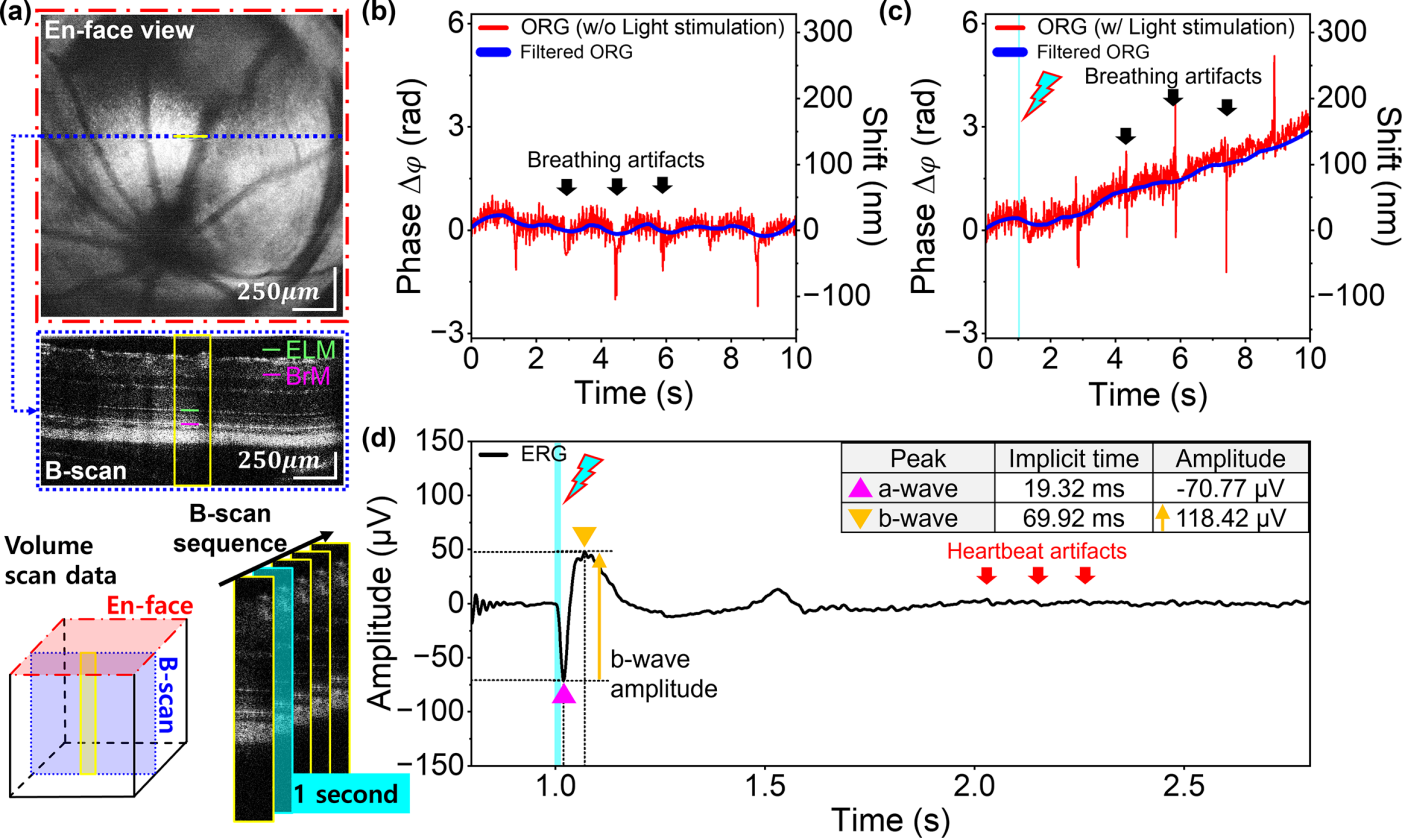
Light-driven ORG and ERG signals with 47.92 µJ for 20% bleaching in WT albino mice. (**a**) BM-scan area corresponding to the *yellow box* and the distance measuring target (BrM-ELM). The parts marked in *light blue* in (**a**), (**c**), and (**d**) represent light stimulation timing. (**b**) ORG signal in the baseline experiment without light stimulation. No significant changes occurred in the phase and shift values between 0 and 10 seconds. (**c**) Light-evoked ORG signals showing relative swelling with an initial phase value of 0. Basic motion correction, using the cross-correlation method, was employed in phase-based ORGs to minimize motion artifacts. However, it was not possible to completely remove significant motion artifacts from heartbeats and breathing. These artifacts depended on the anesthesia states and individual differences between the mice. To provide clear ORG signals, a 0.05- to 60-Hz bandpass filter and a Savitzky-Golay filter were applied. (**d**) ERG graph including a-wave with negative voltage levels and b-wave with positive voltage levels during retina exposure to 482-nm bleach stimuli.

The full-field illuminating LED channel irradiated the mouse eye with 23.96 µJ and 47.92 µJ, corresponding to 10% and 20% bleaching, respectively. Figure 4 presents the representative retinal responses for a 20% bleach level, while those for a 10% bleach level are shown in Supplementary Figure S10. The BM-scan, acquired at a rate of 1 kHz for 10 seconds (Fig. 4a), was used to extract the ORG signals. Figures 4b and 4c show the time-dependent distance changes between the ELM and the BrM using phase-based ΔOPL (optical path length changes) calculations. The initial value of the phase difference was set to zero as a reference, and the graphs displayed the relative change in the distance between the two layers over a 10-second period. Figure 4b shows a lack of ORG signal without any light stimulation as a baseline. Figure 4c displays the standard kinetics of the ORG signal, indicating a bleach-induced swelling of the outer retina. As a result of light stimulation, the ΔOPL distance between the ELM and the BrM has extended ∼152.06 ± 9.46 nm at 10 seconds. The ERG graph in Figure 4d shows the a-wave peak response of the photoreceptors, the b-wave peak response of the bipolar cells, and Müller glial cells. The implicit times of the a-wave and b-wave were the time from the flash to the a-wave trough and b-wave peak. While the amplitudes of the a-wave were measured from 0 µV as a reference, the reference for the b-wave amplitudes was the voltage level of the a-wave trough.

### Light-Evoked ORG and ERG Responses in WT-Pigmented Mice

**Figure 5.**
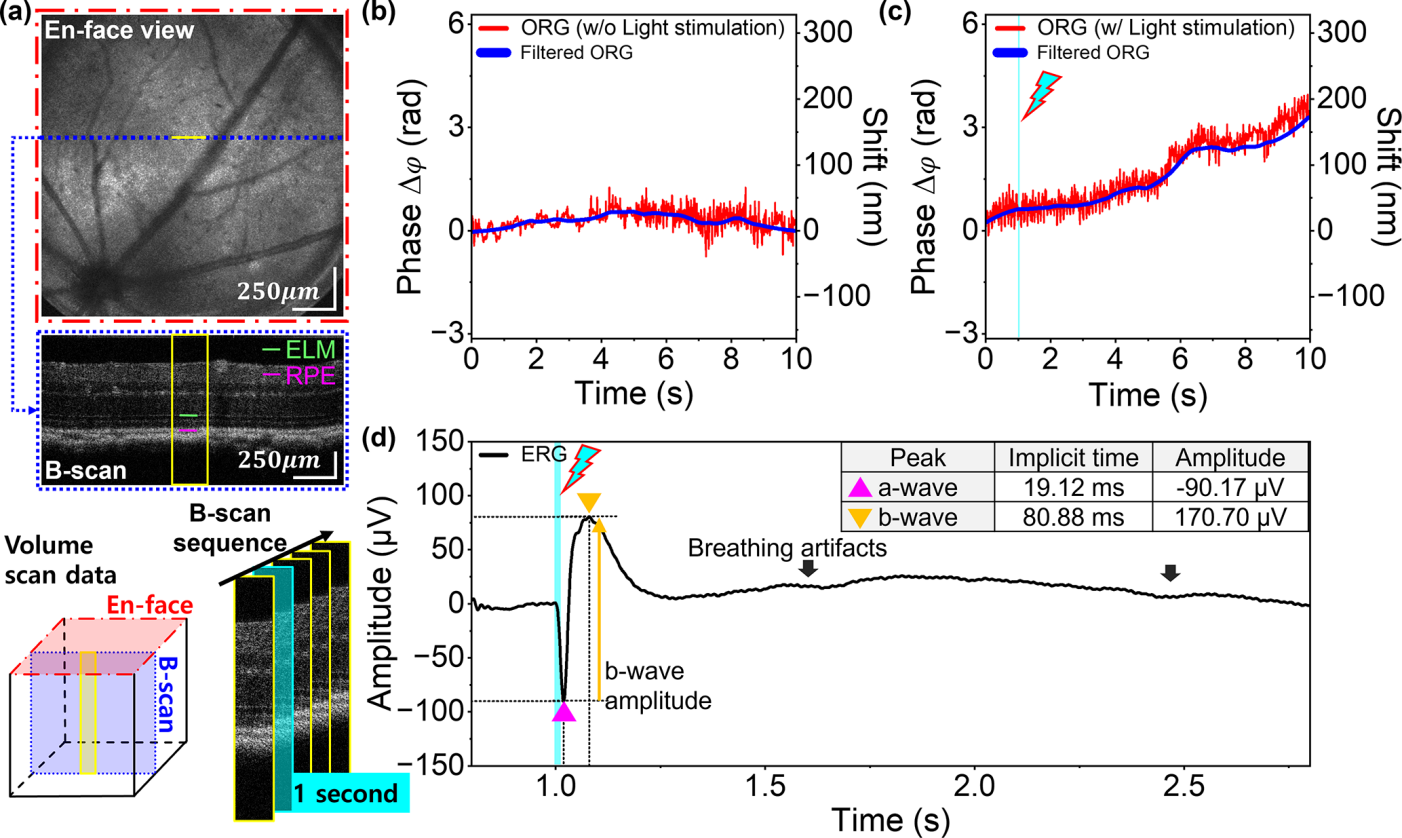
Light-induced ORG and ERG responses with 47.92 µJ light stimulation for 20% bleaching in WT-pigmented mice. (**a**) BM-scan region marked by the *yellow box* and the distance measurements taken between RPE and ELM. (**b**) Baseline ORG signal without light stimulation, showing no significant phase shift throughout the 0- to 10-second interval. (**c**) The light-evoked ORG responses exhibited relative deformation with a phase value of 0 at 0 seconds. (**d**) ERG responses displaying a-wave with negative voltage levels and b-wave with positive voltage levels during retina exposure to 482-nm bleach stimuli.

An experiment was performed using WT-pigmented mice as a normal model to compare with the retina degeneration model. Since melanin was responsible for increased scattering in the RPE and choroid layers, BrM was not detectable in B-scan images. For this reason, RPE was used as an alternative for the reference layer for the ORG signal extraction (RPE-ELM). We irradiated the LED light with 47.92 µJ and 95.84 µJ on the mouse eye, corresponding to 20% and 40% bleach, respectively. Figure 5 and Supplementary Figure S11 presents the representative retinal response for 20% and 40% bleaching, respectively. As in the evaluation of the ORG signals (ELM-BrM) in the albino mice, the ORG signals (ELM-RPE) in Figure 5 exhibited no changes under the baseline condition (without light stimulation), whereas the distance between the ELM and the RPE was extended ∼173.98 ± 9.46 nm until 10 seconds under LED light stimulation. Although the magnitude of elongation could be higher than 174 nm after 10 seconds, the measurements after 10 seconds were limited due to the out-of-memory error in the acquisition session on the computer. The increment of the noise variance in Figure 5b might be from random phase noise.

### Light-Evoked ORG and ERG Responses in Pigmented rd10 Mice

**Figure 6.**
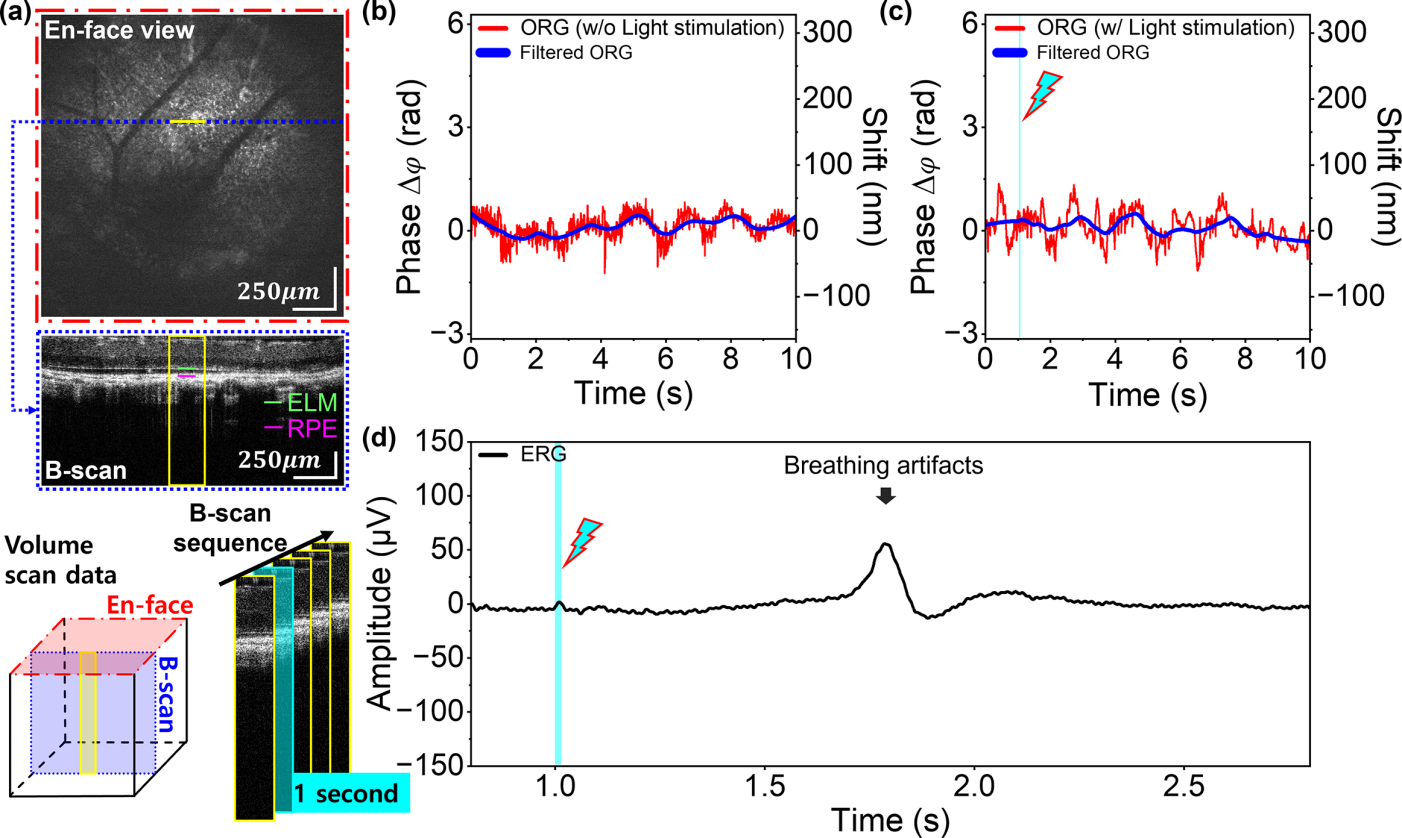
Light-driven ORG and ERG signals with 95.84 µJ light irradiation for 40% bleaching in rd10 mice. (**a**) BM-scan area corresponding to the *yellow box* and the distance measuring target (RPE-ELM). The parts marked in *light blue* in (**a**), (**c**), and (**d**) denote the light stimulation. (**b**) ORG signal for baseline without light stimulation. Since the rd10 models already showed damage in the outer retinal layers, including the photoreceptor layers, the B-scan in (**a**) showed a thinner retina than those in Figures 4a and 5a. (**c**) ORG signal with light stimulation. The ORG signals in (**b**) and (**c**) showed minor fluctuations, which cannot be attributed to light irradiation effects. To provide clear ORG signals, a 0.05- to 60-Hz bandpass filter and a Savitzky-Golay filter were applied. (**d**) The ERG graph showed only a pulse-wave response resembling breathing artifacts without any a-wave or b-wave responses. In Supplementary Figure S13b, the extended ERG signal presented the repeated pulse wave caused by breathing artifacts.

As expected, despite following the same imaging and light stimulation protocols for the retinal degeneration mouse model, ORG signals indicating elongation of the outer retinal layers (ELM-RPE) were not observed. Figure 6 displays the retinal responses of the pigmented rd10 mouse to visible light energy for 40% bleaching (95.84 µJ) and the 20% bleach level responses are available in Supplementary Figure S12. As seen in the B-scan image in Figure 6a (blue-dotted line), the outer retina (ELM-RPE) was significantly thinned in the disease model. The ERG measurement also failed to yield recordings of the a-wave and b-wave peaks. The lack of ORG and ERG signals indicated that the phototransduction in the retina was not present, which was also confirmed by the structural atrophy of the photoreceptors shown in the B-scan in Figure 6a.

### Comparison of ORG and Full-Field ERG Signal Dependence on Bleach Levels

**Figure 7.**
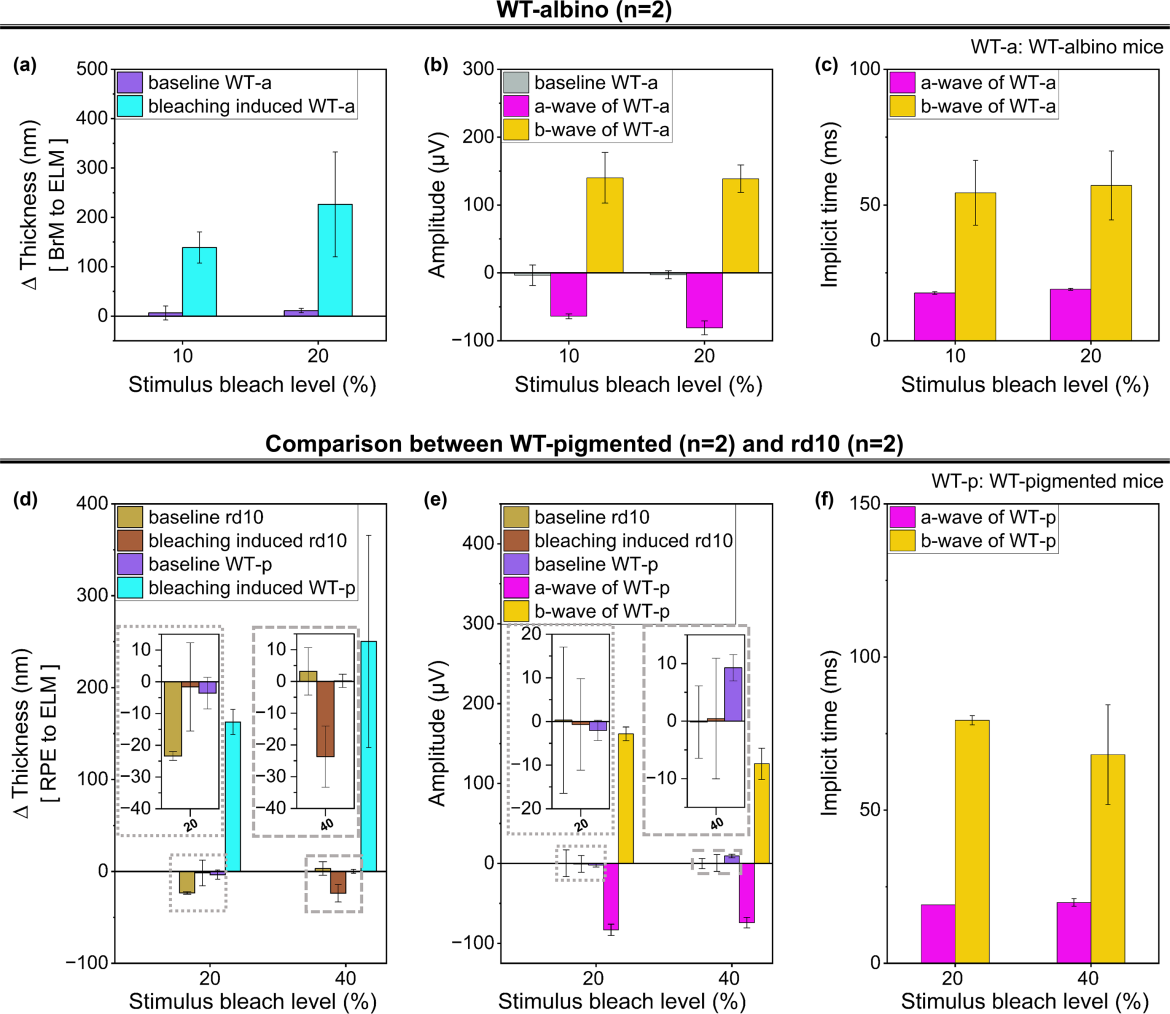
Summary of ORG and ERG responses measured in WT-albino, WT-pigmented, and rd10 mice depending on light stimulus bleach levels (*n* = 2 per group). (**a**) Maximum swelling of the outer retina in WT-albino mice in responses to 10% and 20% bleach levels and corresponding baseline (0% bleach level), respectively. (**b**) ERG amplitudes of the a- and b-waves for the same two stimulus bleach levels in (**a**) and baseline ERG responses (no ERG signal amplitude). (**c**) Comparison of implicit times of a- and b-waves induced by two stimulus bleach levels (no waves for baseline measurements). (**d**) Thickness changes due to 20% and 40% stimulus bleach levels in rd10 and WT-pigmented mice. Similar to the albino experiments shown in (**a**), the thickness difference between the RPE and the ELM was negligible for the baseline experiments (0% bleach level). *Inset* depicts magnified small values of ∆Thickness depending on stimulus bleach levels. (**e**) ERG amplitudes of the a- and b-waves for the same two stimulus bleach levels in (**d**) and baseline ERG responses (0% bleach level) for rd10 (no waveform was observed in ERG responses), as well as WT-pigmented mice. *Inset* shows zoomed-in small-amplitude values in ERG signals depending on stimulus bleach levels. (**f**) Comparison of implicit times of a- and b-waves induced by two stimulus bleach levels (no waves for rd10 mice and baseline measurements). *Error bars* represent standard deviation. ORG responses in (**a**) and (**d**) show a bleach level–dependent tendency in swelling amplitude. ERG responses in (**b**), (**c**), (**e**), and (**f**) do not show bleach level–dependent changes. The differences between baseline and stimulus-driven rd10 experiments were not statistically significant based on the XY statistical test.

The visible light stimulations in the WT mice resulted in a proportionally increasing tendency in the distance between the BrM and the ELM, as well as between the RPE and the ELM, depending on the energy of the different bleach levels, as shown in Figures 7a and 7d. While the baselines (without light stimulus) in the graphs showed slight thickness changes over 10 seconds, these changes did not exhibit a continuous increase or decrease throughout acquisition. The constantly changing values without specific patterns were assumed to originate from motion artifacts and phase instability. Although the ORG exhibited a proportional relationship with the bleach energy, the implicit times and amplitudes of the ERG were not influenced by the bleach levels to the same degree. In our experiments, the bleach levels were varied by adjusting the duration of irradiation with a fixed light intensity. The light power was considered a crucial factor affecting the characteristics of ERG peaks because these peaks have similar implicit times and amplitudes. These aspects were observed for both the albino and pigmented mice. For the rd10 mice, the thickness increase in the retinal layers was not observed after light stimuli, and ERG signals could not be obtained due to the damaged neural function of the retina.

## Discussion

ORG measured the light-evoked morphologic changes in outer retinal layers (ELM-BrM or ELM-RPE) during serial OCT imaging. To quantify the retinal layer deformation, we have previously conducted intensity-based and phase-based ORG studies.^2^^,^^16^ Intensity-based structure deformation measurements have limitations in quantifying minute shifts in deformation. Still, measurements of deformations smaller than the axial resolution, down to tens of nanometers, were possible for well-defined layers (high SNR) seen on OCT. The phase-based ORG processing had the advantage of detecting nanoscale thickness changes between any two arbitrary positions within the A-scan. The high noise level of the ORG phase-based data shown here could lead to a small error in the actual path length difference due to heartbeat and breathing variations. As shown recently by Tan et al.,^9^ alternative processing methods should be explored to reduce the noise of the measured ORG signals. In addition, improvement for real-time motion correction based on reference tracking might reduce the noise of the ORG signal by motion variations during data acquisition steps. Individual mouse ORG and ERG results showed different levels of noise from breathing and heartbeat, but real-time motion correction will mitigate these artifact levels.

The advancement achieved in the present study included simultaneous ERG measurement and full-field flashlight stimulation. The LED illumination channel did not require scanning, unlike the system used in our previous study,^2^^,^^16^ which required raster scanning of the bleaching beam. Here, we simultaneously acquired ERG signals and serial B-scans during visible light stimulation. The light energies for each stimulation were modulated by adjusting the exposure duration while maintaining a fixed light intensity. Since adjusting light stimulus energy by exposure time did not conform to the International Society for Clinical Electrophysiology of Vision guideline, a higher-power light source combined with optical attenuation (neutral density filter sets) should be explored for future experiments on ERG-integrated ORG. This would allow changing the bleach level without changing the exposure time. Additionally, our current OCT system's sensitivity did not allow measurement of ORG responses at low bleach levels, often used in ERG studies.

Through postprocessing via phase-based quantification of ΔOPL on the BM-scans and ERG data extraction, we obtained ORG and ERG graphs revealing the bleach level–dependent responses of the retina, including increased retinal thickness as well as a- and b-waves of ERG. The ORG results from experiments using albino and pigmented WT mice revealed, as expected based on the bleach level–dependent ORG by Tan et al.,^9^ a proportional increasing tendency in retinal layer thickness with bleach light levels. As presented in Figures 7a and 7d, when the energy level was doubled (bleach level 20% in WT-albino and 40% in WT-pigmented), the average increase in thickness reached 168% and 147% respectively, compared to half of each bleach level (10% in WT-albino and 20% in WT-pigmented). Baker et al.^35^ reported that rod bipolar cells were significantly reduced in albino retinas compared with pigmented rats. Low rod numerosity could be assumed to have low sensitivity and low light-evoked response corresponding to the amplitude of peaks of ERG signals, but the albino rabbit was more sensitive than the pigmented rabbit reported by Ioshimoto et al.^36^ According to this ERG comparison, the high sensitivity of albino rabbits might be attributed to greater availability of light due to scatter and reflection at the retinal layer. We expected the high sensitivity of the albino model to affect a higher average increase ratio than pigmented models. Although we indirectly compared ORG/ERG results by observing a doubling of stimulus energy level between albino and pigmented mice here, retinal sensitivity and rod numerosity-dependent ORG, combined with ERG, will be a critical approach to understanding ORG physiology in our future work. While WT albino and pigmented mice showed bleach level–dependent ERG and ORG responses, including the proportionally increasing tendency, no reactions arose in the rd10 mice, as shown in Figures 7d and 7f. Since thinned and damaged photoreceptor layers were observed with rd10 mice from the volume-scanning procedure, the aforementioned lack of light-evoked responses was caused by the absence of activation for the phototransduction process due to the damaged photoreceptors and the death of most of these neurons. These tendencies confirm that ORG signals can be acquired and analyzed to assess the remaining neural function in partially damaged retinas in animal models. The nonresponsive ORGs and ERGs in the completely photoreceptor-degenerative rd10 mice (3 months of age) validated the feasibility of the ORG system for screening between normal and blind animals. However, further studies would be needed to assess system sensitivity to grade disease progression in retinal degeneration. We plan to investigate ORG and ERG responses in retinal degeneration at each disease stage of rd10 (from predegeneration to rod degeneration completion) in an appropriate sample size in the future. Additionally, retinal conditions in all mice models should be cross-validated via histologic analysis.

Whereas the ORG data in each experiment exhibited a bleach level–dependent tendency in swelling amplitude, the characteristic differences in each peak for the a- and b-waves were not significant with respect to the bleach levels. We presumed that varying the light exposure conditions by adjusting the exposure duration was not suitable for inducing ERG signals with varying implicit times and amplitudes. This was because the implicit times and amplitudes of each wave peak depended on the illumination intensity, as observed in previous ERG studies.^37^^,^^38^ Additionally, for the pigmented WT mice, 40% bleach led to shorter implicit times and lower amplitudes than 20% bleaching. Although the reason for this observation was unclear, we believed that lighting durations longer than 50 ms allowed the retina to adapt to light exposure and reduce its response. For example, clinical research has reported that after 20 ms of exposure, the electrical signals were split as the b-wave and d-wave of human ERG, and the b-wave amplitudes decreased.^37^ Thus, intensity-dependent light stimulation for 5 ms will be considered in future studies to compare bleach-driven ORG and ERG responses. The relatively low amplitude of the full-field ERG response is probably due to the nonoptimal design of the corneal electrode and the limited retinal area being stimulated. We will try to address these issues in the future. Additionally, ORG signals could be extracted from any two retinal layers located outside of the outer segment, not just ELM-BrM, as shown in this study. Although the ORGs of the ELM-RPE layer and the ELM-BrM were not a perfect substitute for each other, they were enough to show elongation of the outer segments. If the RPE layer also exhibited significant morphologic alterations in response to light stimulation, then selecting RPE and BrM boundaries for ORG calculation would be crucial for ORG comparison. However, distinct morphologic changes in RPE were observed after exposure for ∼1.5 hours at 6000 lux, which involved a much higher light energy than our stimulus protocol.^39^

Unlike ERG, which showed rapid responses within 100 ms after light stimulation, ORG showed much longer responses lasting over 10 seconds. In comparison, the electrical signals of ERG originated from the immediate electrical responses of photoreceptors, bipolar cells, and Müller glial cells. The source of the light-driven rise in osmotic pressure (water activity) between the photoreceptor's OS and the subretinal space was the light activation of rhodopsin and the resulting phototransduction cascade.^16^^,^^18^^,^^40^^–^^42^ We expected the ORG signals (swelling of OS) to increase until osmotic pressure equilibrium was reached and then decrease once the effects of phototransduction were entirely reversed. The primary role of water movement in causing retinal swelling and scattering changes has recently been confirmed.^12^ Since the osmotic equilibration and regeneration process after light stimulation required a significantly longer time (>100 seconds) than the rapid electrical responses, such as the a-wave (∼20 ms) and b-wave (∼100 ms) in ERG, the ORG signal was measured longer than the ERG signals.^16^ The results of much longer ORG responses than ERG responses were also observed in another ORG-ERG combined study in humans by Dhaliwal et al.^43^ and in mice by Tan et al.,^9^ suggesting a possible link with the c-wave. Supplementary Figure S1 describes the sequence of light-evoked responses in the outer retina and the possible mechanistic chain of events responsible for ORG responses observed over a longer time than the a- and b-waves of ERGs, as it was linked with osmotic equilibration rather than the electrical cellular responses.

Combining ORG with ERG in the mouse model was a valuable initial step for understanding ORG origins and the physiology of phototransduction via light stimulation. Comparisons between baseline and rd10 models suggested the feasibility of developed OCT for ORG to detect photoreceptor loss. To translate ORG + ERG into human studies, key technical challenges remained (e.g., phase stability, real-time motion correction, correlation between the ORG signal and the numerosity of photoreceptors, and optimizing the light stimulation protocol). Once these advances have been achieved with the ORG technology, ORG with ERG could be applied to a potential clinical investigation for early diagnosis of photoreceptor dysfunction. Ultimately, we expect that advanced ORG, combined with ERG, will provide ORG mapping of retina, similar to multifocal ERG, to reveal photoreceptor function across the retina.

## Supplementary Material

Supplement 1
