# Supplementary material for: Combined Mouse Retinal Optoretinography/Electroretinography System to Study Light-Evoked Responses in Animal Models of Retinal Degeneration
Source: Invest Ophthalmol Vis Sci. 2026 Jan 20;67(1):39. doi: 10.1167/iovs.67.1.39 (PMC12831139; doi:10.1167/iovs.67.1.39)
Supplement: Supplement 1 [file iovs-67-1-39_s001.docx]

*For Investigative Ophthalmology & Visual Science*

Supplementary Information - Combined mouse retinal optoretinography (ORG) / electroretinography (ERG) system to study light-evoked responses in animal models of retinal degeneration.

Hang Chan Jo^1,2,3,4^, Ewelina A. Pijewska^2,3,5^, Ratheesh K Meleppat,^2,3,4^ Ravi S. Jonnal^3,4^, Ala Moshiri^4^, Dae Yu Kim^1,2,3,4^, and Robert J. Zawadzki^2,3,4^*

^1^ Department of Electrical and Computer Engineering, College of Engineering, Inha University, Incheon, Republic of Korea

^2^ UC Davis EyePod Small Animal Ocular Imaging Laboratory, Department of Cell Biology and Human Anatomy, University of California Davis, Davis, CA, United States of America

^3^ Center for Human Ophthalmic Imaging Research (CHOIR), UC Davis Eye Center, Department of Ophthalmology and Vision Science, University of California Davis, Sacramento, CA, United States of America

^4^ Department of Ophthalmology and Vision Science, University of California Davis, Sacramento, CA, United States of America

^5^ Institute of Physics, Faculty of Physics, Astronomy and Informatics, Nicolaus Copernicus University, Toruń, Poland

# Section 1. Light evoked water movement


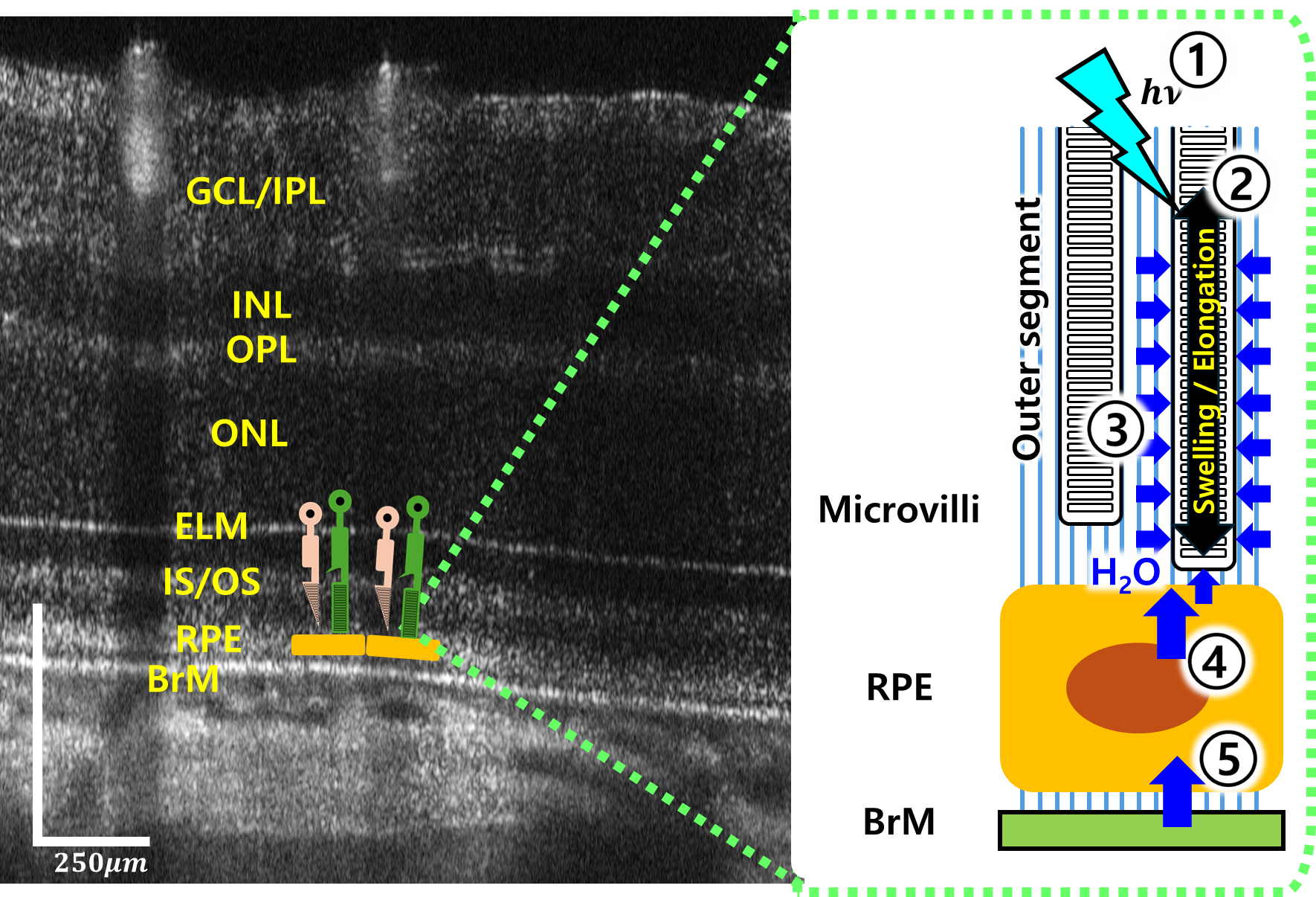


**Fig. S1**. The scheme of illustrating the process of light-evoked response in outer segment (OS)-retina pigment epithelium (RPE)-Bruch’s membrane (BrM) complex. Individual numbers represented the order of the responses. Elongation of the OS occurred in the third process. ①light stimulation, ② charge-dependent disc membrane force change including hyperpolarization and depolarization, energy source for ERG signal, ③, ④, and ⑤ water movement caused by osmotic pressure changes.^12^

# Section 2. Custom-built OCT / ORG + ERG system

We built a spectral-domain optical coherence tomography (OCT) system to image the mouse retina for optoretinogram (ORG) processing. The OCT system was combined with a commercial electroretinogram (ERG) system as a reference to investigate ORG physiology. Fig. S1 shows a photograph of the developed system during in vivo mouse imaging and ERG measurements.


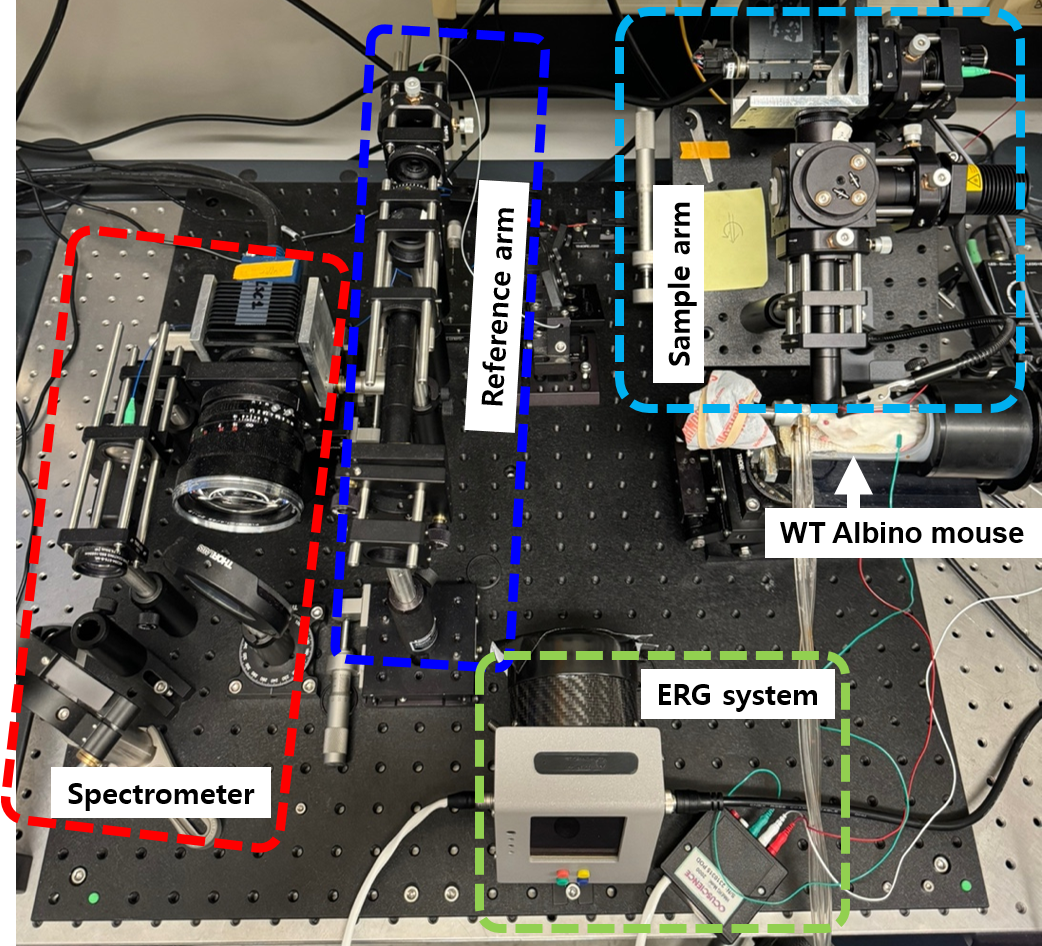


Fig. S2 Photograph of the developed OCT / ORG + ERG system. The OCT system comprised a spectrometer, reference arm, and sample arm. The spectrometer included a line scan camera (SpL4096-140km, Basler, Ahrensburg, SH, Germany) and an infrared-optimized lens (The Planar T*1.4/85 ZF-IR, Carl Zeiss, Oberkochen, BW, Germany). The reference arm and sample arm consisted of the same optical components, i.e., achromatic doublet lenses, except for the contact lens and beam splitter for the LED stimulation channel. The manufacturer of the ERG system restricted its light source via circuit breaking and blocked the outlet for the light with black duct tape as well. The ERG system automatically adjusts the display's brightness in a dark room.

# Section 3. Estimation of bleach light field illumination angle

Complementary metal-oxide semiconductors (CMOSs) are integrated circuits that sense photons for imaging in a camera module. If the CMOS sensor captures the beam on its surface, the acquired data will show the intensity profile of the beam. A custom program was developed to extract the full-width-at-half-maximum (FWHM) of the beam intensity profile, which represented the beam diameter. The software calculated the diameter of visible LED beams based on the distance between the end of the imaging probe and the CMOS sensor (0.0–3.0 mm). The linear stage of the imaging probe was used to adjust the distance during beam capture. Assuming the axial length of the mouse eye to be 3.0 mm and disregarding the refraction between the cornea and the lens of the mouse eye, the field illumination angle of the bleach light was ~$52.8^{\circ}$.


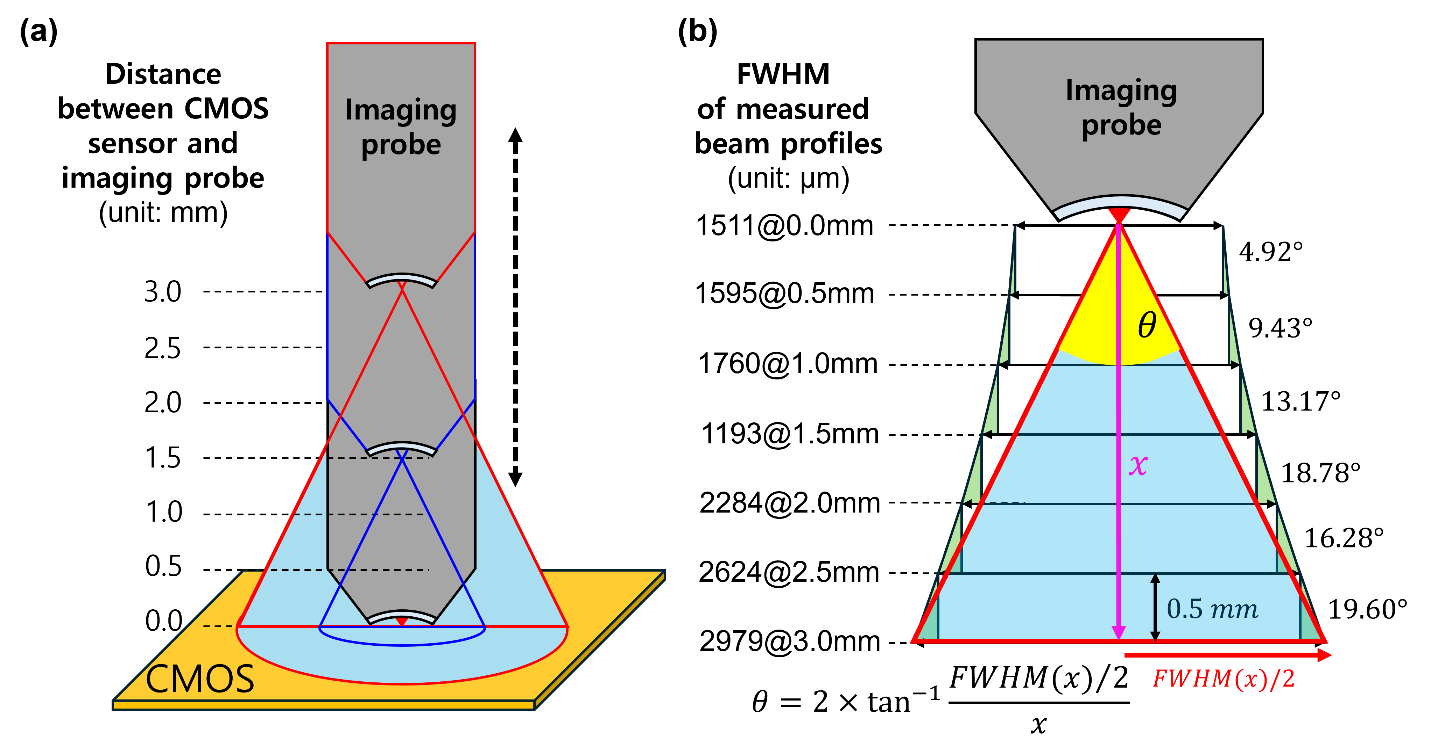


Fig. S3 Beam profiling process: (a) schematic of the CMOS sensor capturing the LED beam based on the distance between the sensor and the imaging probe; (b) FWHM-based calculation of the light illumination angle. In this process, the beam size at the distance of 0.0 mm (i.e., the focal plane of the beam) was considered 0 µm.

# Section 4. Estimation of illumination area on the retina surface

The field-illuminated area on the retina was calculated based on the measured illumination angle of ~$52.8^{\circ}$ (Supplementary 2). If the estimated visual angle $\theta_{v}$ was $52.8^{\circ}$, the estimated eye angle $\theta_{e}$ would be $79.2^{\circ}$, which is 1.5 times $\theta_{v}$^29^. The formula for the side surface area of a solid of revolution could be used to estimate the area:

$\begin{aligned} S=\int_{a}^{b} 2\pi f\left( x \right)\sqrt{1+\left\{ f^{'}\left( x \right) \right\}^{2}}dx.\#\left( 1 \right) \end{aligned}$Considering the eyeball to be a perfect sphere with radius r, the exposed area on the retina was delineated by the light green region in Fig. S3(b), and $f\left( x \right)$ is calculated as follows:

$$\begin{aligned} f\left( x \right)=\sqrt{r^{2}-x^{2}}.\#\left( 2 \right) \end{aligned}$$

Based on Eq. (1), the field illumination was estimated as follows:

$$\begin{aligned} S=\int_{-r}^{r} 2\pi\sqrt{r^{2}-x^{2}}\sqrt{1+\left\{ \frac{r\frac{dr}{dx}-x}{\sqrt{r^{2}-x^{2}}} \right\}^{2}}dx.\#\left( 3 \right) \end{aligned}$$

Since the radius r was a constant, the simplified equation for the surface area of a sphere was

$$\begin{aligned} S=\int_{-r}^{r} 2\pi rdx.\#\left( 4 \right) \end{aligned}$$

The radius r was the same as $l`$ in Fig. S3(a), which was approximately 1.680 as calculated based on the following proportionality:

$$\begin{aligned} l :l`=\tan\left( \frac{\pi}{2}-\frac{\theta_{v}}{2} \right):\tan\left( \frac{\pi}{2}-\frac{{1.5\theta}_{v}}{2} \right)=\cot\frac{\theta_{v}}{2}:\cot\frac{{1.5\theta}_{v}}{2}.\#\left( 5 \right) \end{aligned}$$

Using equation (4) and the radius calculated based on the proportionality above, the exposed area on the retina was estimated:

$$\begin{aligned} \int_{r\cos\frac{\theta_{e}}{2}}^{r} 2\pi rdx={2\pi r\left[ x \right]}_{0.229}^{1.680}=4.061 {mm}^{2}.\#\left( 6 \right) \end{aligned}$$

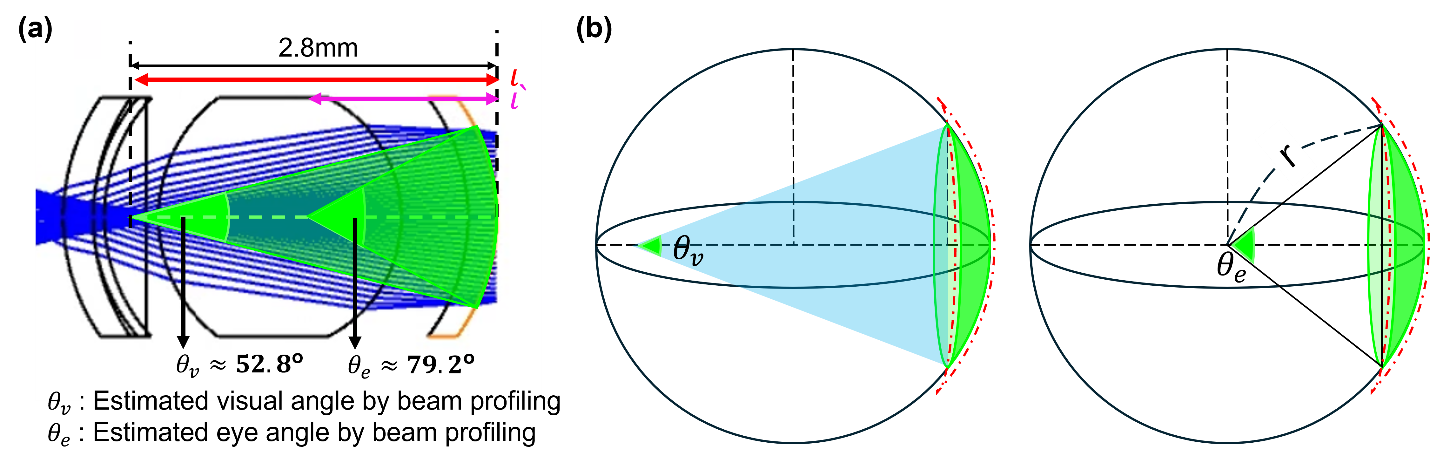


Fig. S4 Schematic illustrating the light-exposed area with the illumination angle: (a) optical simulation with estimated visual and eye angle; (b) each angle and radius in the case of the sphere.

# Section 5. Phase stability of the OCT system for ORG signal extraction

Phase-based ORG processing compared the temporal changes in phase differences between two specific layers, e.g., the external limiting membrane (ELM) and Bruch’s membrane (BrM) of the mouse retina. This processing required stable phase information in sequential B-scans to detect time-dependent changes. To evaluate the stability of the phase from sequential data acquisition, we performed scanning of a glass slide with two well-defined layers representing the surfaces on both sides. After processing the data via a fast Fourier transform, the separated phase values from complex OCT data were expressed as wrapped phase values, ranging from −π to +π. Due to the expression range being limited to –3.14… to +3.14… rad, the phase exhibits spikes in the graph in Fig. S4(a). These spikes led to substantial errors in analyzing the phase differences because an unwrapped phase of ~8.28… is equivalent to a wrapped phase of 2 (2 + 2π). The solution to this problem was unwrapping the phase of all the pixels in the data. The phase differences in a series of B-scans were shown in Fig. S4(a). The theoretical phase error between two layers of a B-scan image in Fig. S4(a) was calculated as 0.13 rad considering SNR (58.54dB). Actual phase instability in the retina tissue was 0.182 rad considering a lower SNR (29.98dB) than the glass plate image.


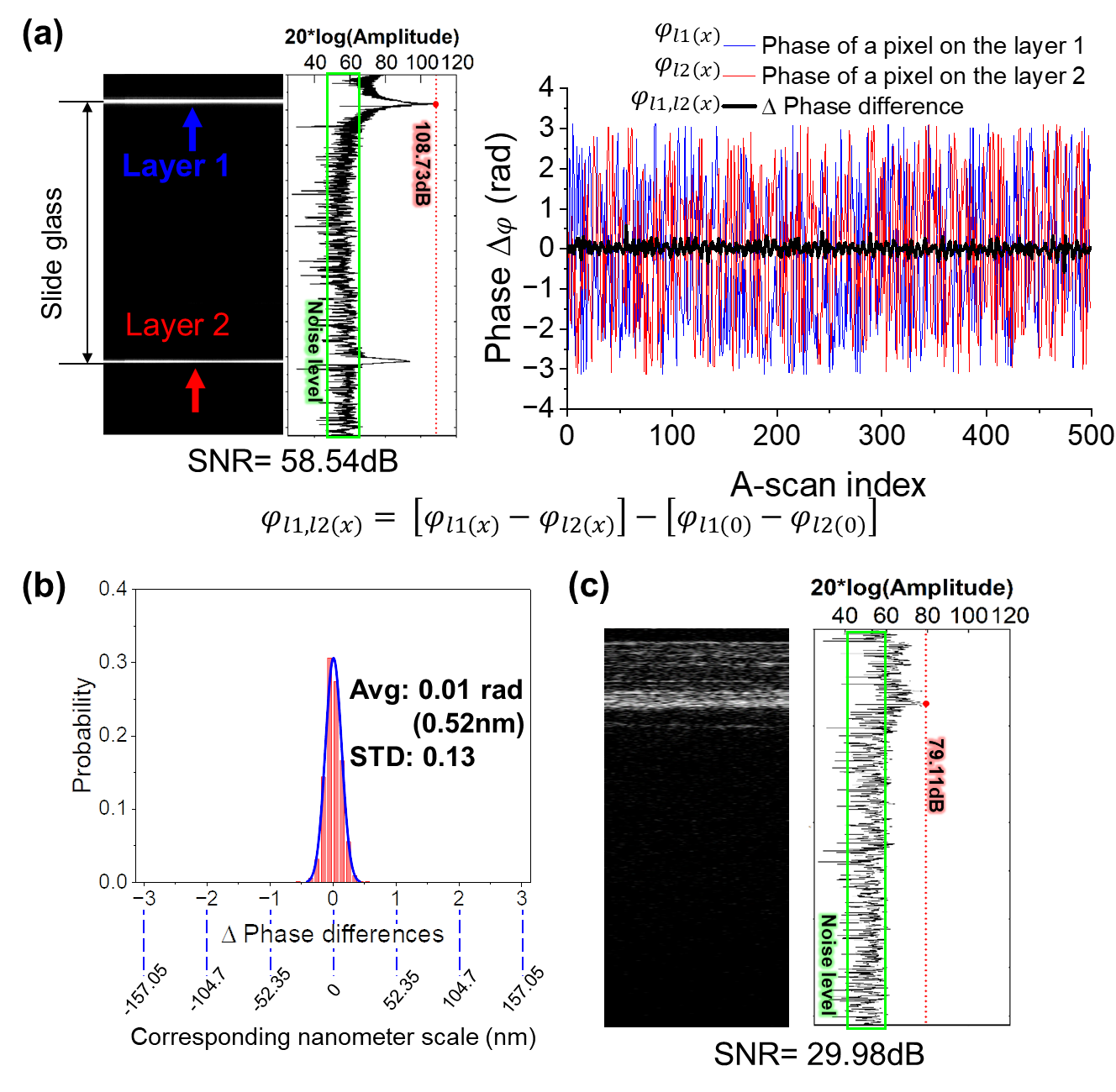


**Fig. S5** Phase stability of ORG experiements based on time-dependent phase difference: (a) B-scan of glass plate in a BM-scan showing Layers 1 and 2 in an intensity image of phase data (left), phase difference, and phase value of each pixel on the layers. Signal to ratio (SNR) of the glass plate image was 58.54dB. (b) Histogram of phase differences over 500 B-scans. The average change in phase differences was 0.01 rad, which corresponded to 0.52 nm based on the time-dependent layer displacement formula. (C) B-scan of mouse retina for comparing SNR to calculate actual phase instability. SNR of the mouse retina image was 29.98dB. According to established phase noise theory by Y Ling, et al. and S Moon, et al. phase stability had a linear relationship with $1/\sqrt{SNR}$ which suggested phase instability of our OCT system in the retina would be approximately 0.182rad $(0.13 rad\times\sqrt{58.54dB}/\sqrt{29.98dB})$.^44,45^

# Section 6. B-scan image comparison between albino and pigmented mice


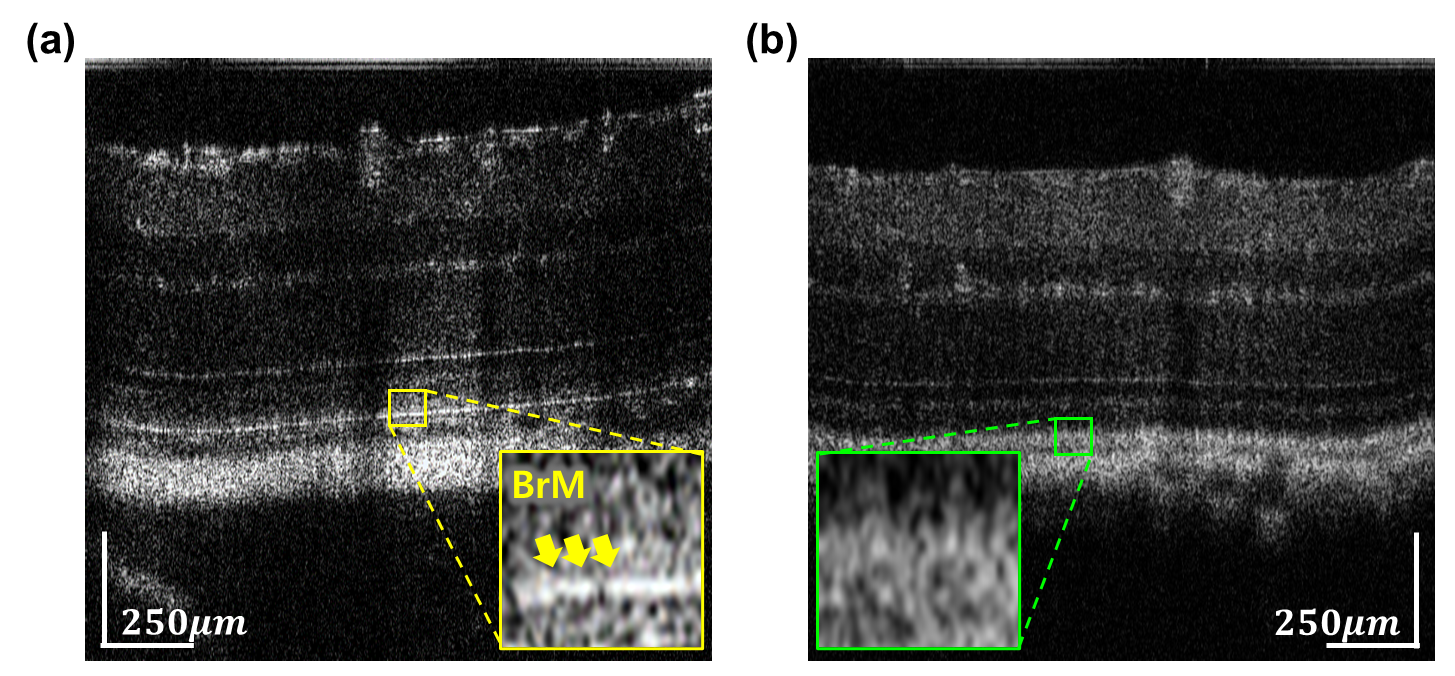


**Fig. S6** The comparison of B-scan images for retina between WT albino and pigmented mice. (a) A retina image of albino mice. (b) A retina image of pigmented mice. Each magenta and green box showed outer retina layers. Bruch’s membrane (BrM) was shown as bright line in the albino image but specific structure like shallow line was not detectable in pigmented mice.

# Section 7. B-scan image comparison between pigmented and rd10 mice


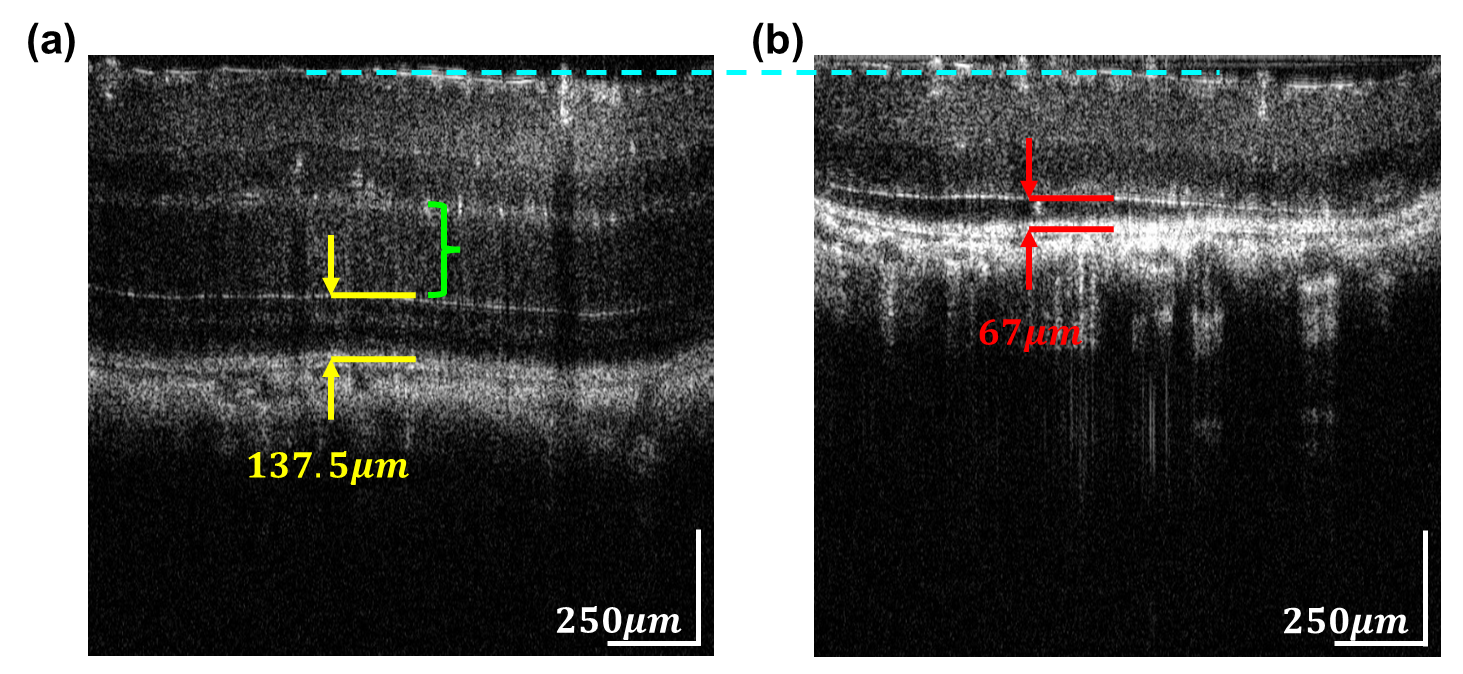


**Fig. S7** The comparison of B-scan images for retina between WT pigmented and rd10 mice. (a) A retina image of pigmented mice. (b) A retina image of rd10. The surface of the individual retina was arranged and marked as a light blue dotted line. Two-line pairs, yellow (a) and red (b), showed decreased thickness corresponding to the distance between the external limiting membrane (ELM) and the retinal pigment epithelium (RPE). The green bracket in (a) highlighted residual ONL thickness which was degenerated in the rd10.

# Section 8. ERG signal processing

The raw signal acquired using the full field-ERG system was processed using a custom signal-processing code in LabVIEW. The signal processing included notch, bandpass, and Gaussian filters. Fig. S5 shows the raw ERG signal and filtered signal after each filtering procedure.


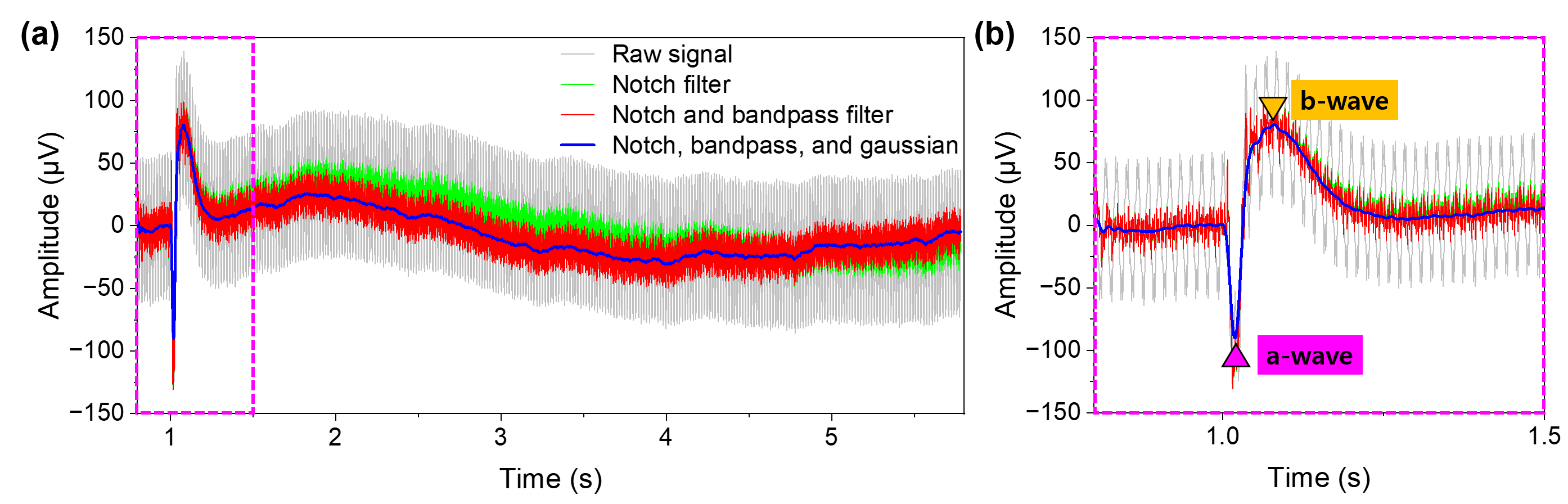


Fig. S8 ERG signal processing results with different filtering combinations: (a) Comparison among all filtered graphs, where the blue graph represents the final ERG signal. (b) Zoomed-in graph for the region delineated by the pink dashed rectangle in (a).

# Section 9. Phase-based ORG processing steps

The BM-scan data of the mouse retina were processed using the phase-based ORG steps reported in our previous work. Phase-based ORG processing enabled improved sensitivity of ORG measurements for morphological changes in the outer retina compared with intensity-based ORG processing. Fig. S6 illustrates the steps of phase-based processing for ORG signals, accompanied by visualized example results. Before this procedure could be executed, we manually segmented two layers of interest, which included the target pixels, for extracting the phase values. The first and second steps involved calculating the temporal difference of the cross-spectrum phase. The representative locations of Steps (1) and (2) are marked in orange and light green in the BM-scan. After calculating the difference corresponding to Step (2) over the range of interest (marked “N”), the phase differences of the cross-spectrum were averaged in Step (3). Steps (4) ~ and (6) involve calculating the weight value to minimize the abnormal data region caused by artifacts related to breathing and heartbeat.


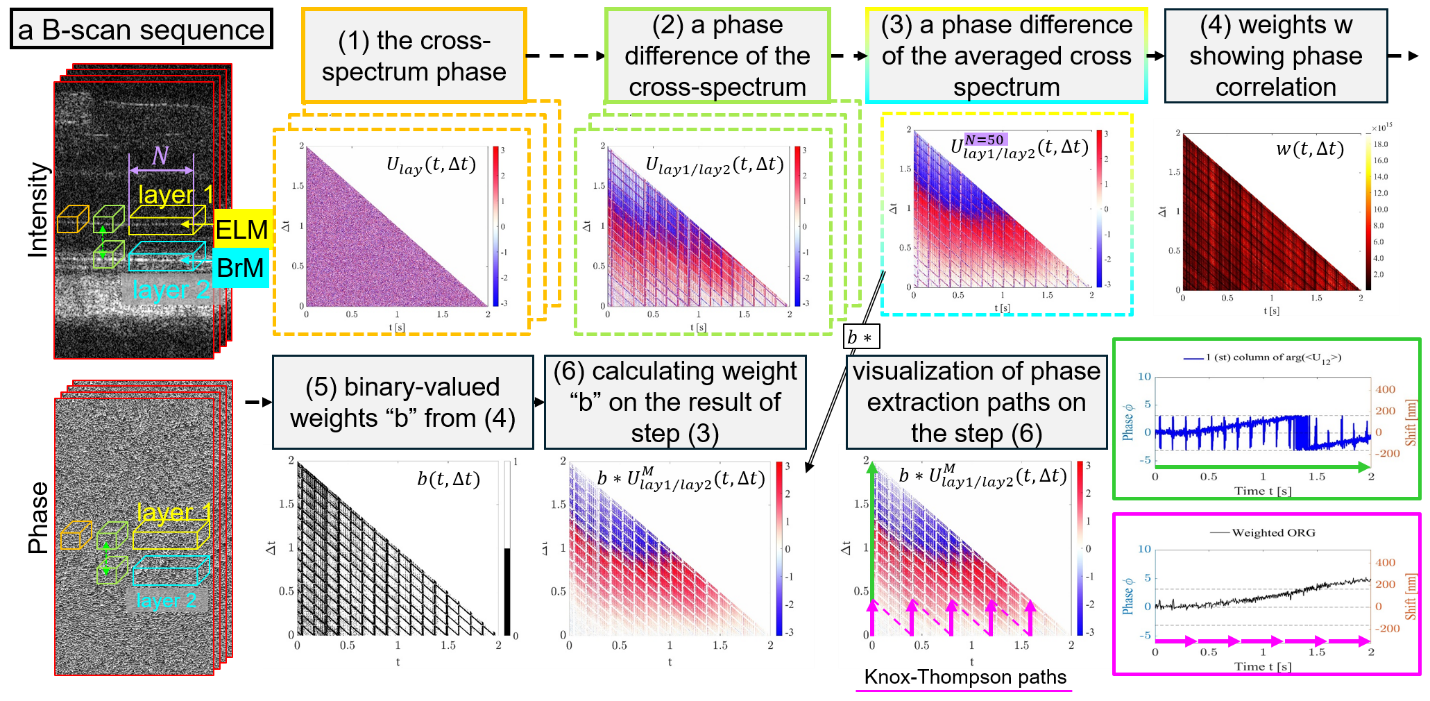


Fig. S9 Outline of phase-based ORG signal extraction with intermediate results reproduced based on the previous work by Pijewska et al^2^.

# Section 10. Light stimulation experiments

Figs. S7, S8, and S9 show additional light-driven ORG and ERG data acquired for different bleach levels. Figs. 7. presents the comprehensive results, which include the data in these measurements.


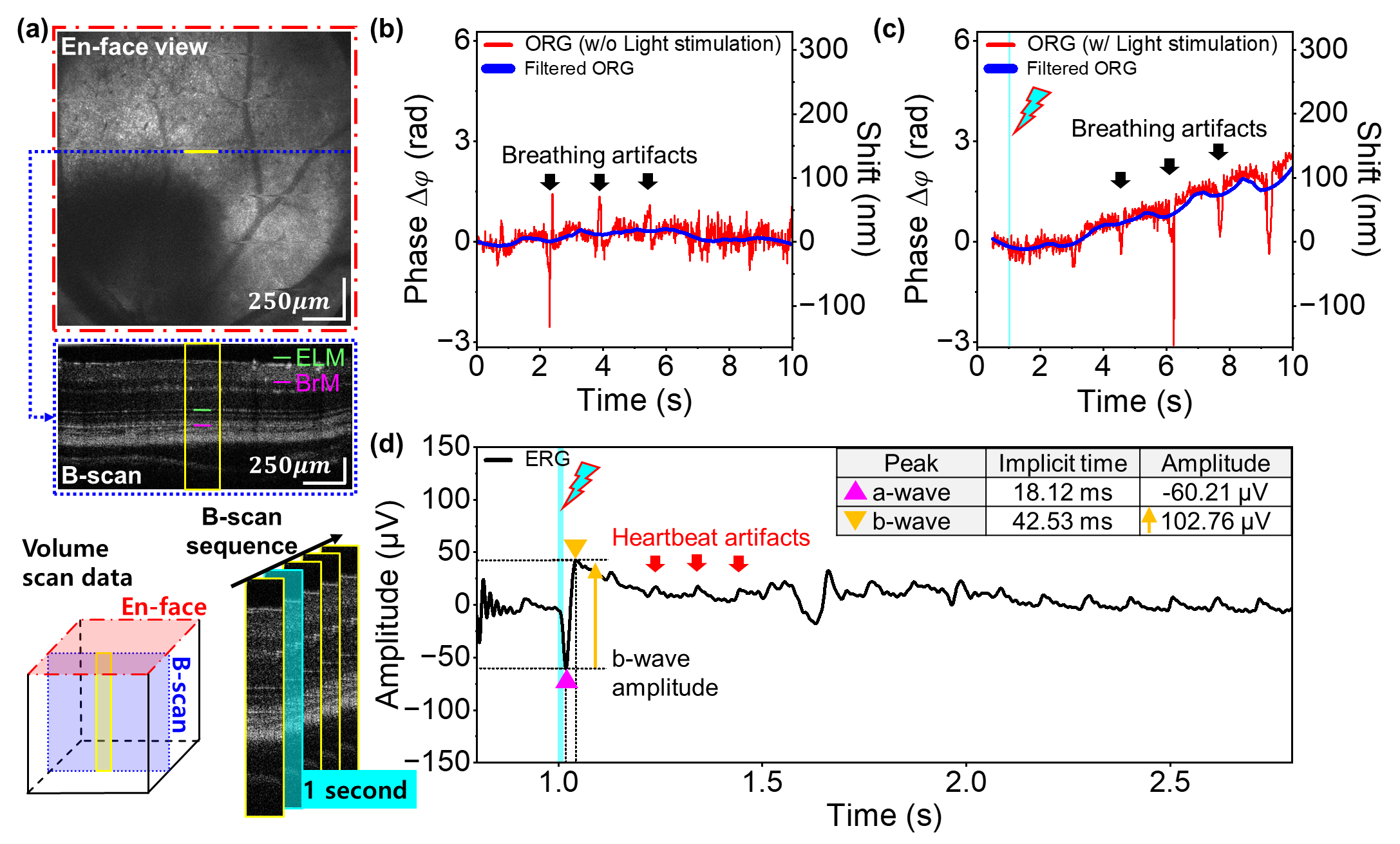


Fig. S10 Light-driven ORG and ERG signals with $23.96 \mu J$ for 10% bleaching in wild-type albino mouse. (a) BM-scan area corresponding to the yellow box and the distance measurements between two layers (BrM to ELM). The parts marked in light blue in (a), (c), and (d) denote the light stimulation. (b) ORG signal for control without light stimulation. No significant changes occurred in the phase and shift values between 0 s and 10 s. (c) Light-evoked ORG signal showing relative elongation with a phase value of 0 s. Since motion correction was not required to process phase-based ORG, the ORG signals included some breathing and heartbeat artifacts, similar to the ERG graphs. These artifacts depended on the anesthesia states and individual differences between the experiments. To provide clear ORG signals, 0.05 – 60Hz bandpass filter and Savitzky-Golay filter were applied. (d) ERG graph including a-wave with negative voltage levels and b-wave with positive voltage levels after the retina was exposed to 482 nm light.


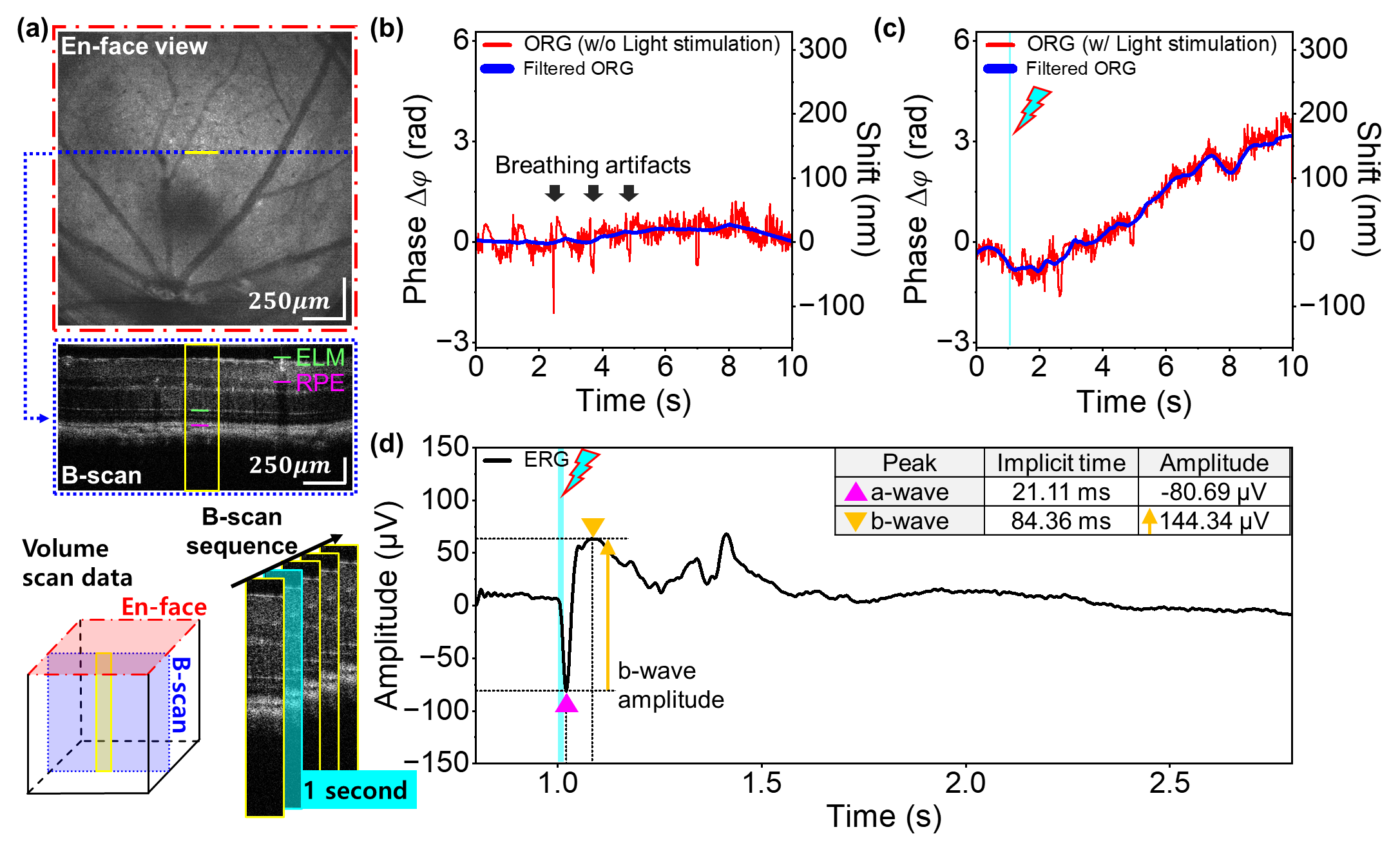


Fig. S11 Light-driven ORG and ERG signals with $95.84 \mu J$ for 40% bleaching in wild-type pigmented mouse. These experiments were the controls for the retinal disease model. (a) BM-scan area and distance measuring target (RPE to ELM). The parts marked in light blue in (a), (c), and (d) represented the light stimulation timing. (b) ORG signal for control without light stimulation. No significant changes occurred in the phase and shift values between 0 s and 10 s. (c) light-evoked ORG signal showing relative deformation with a phase value of 0 s. To provide clear ORG signals, 0.05 – 60Hz bandpass filter and Savitzky-Golay filter were applied. (d) ERG graph showing light-driven electrical response after the retina was exposed to visible light.


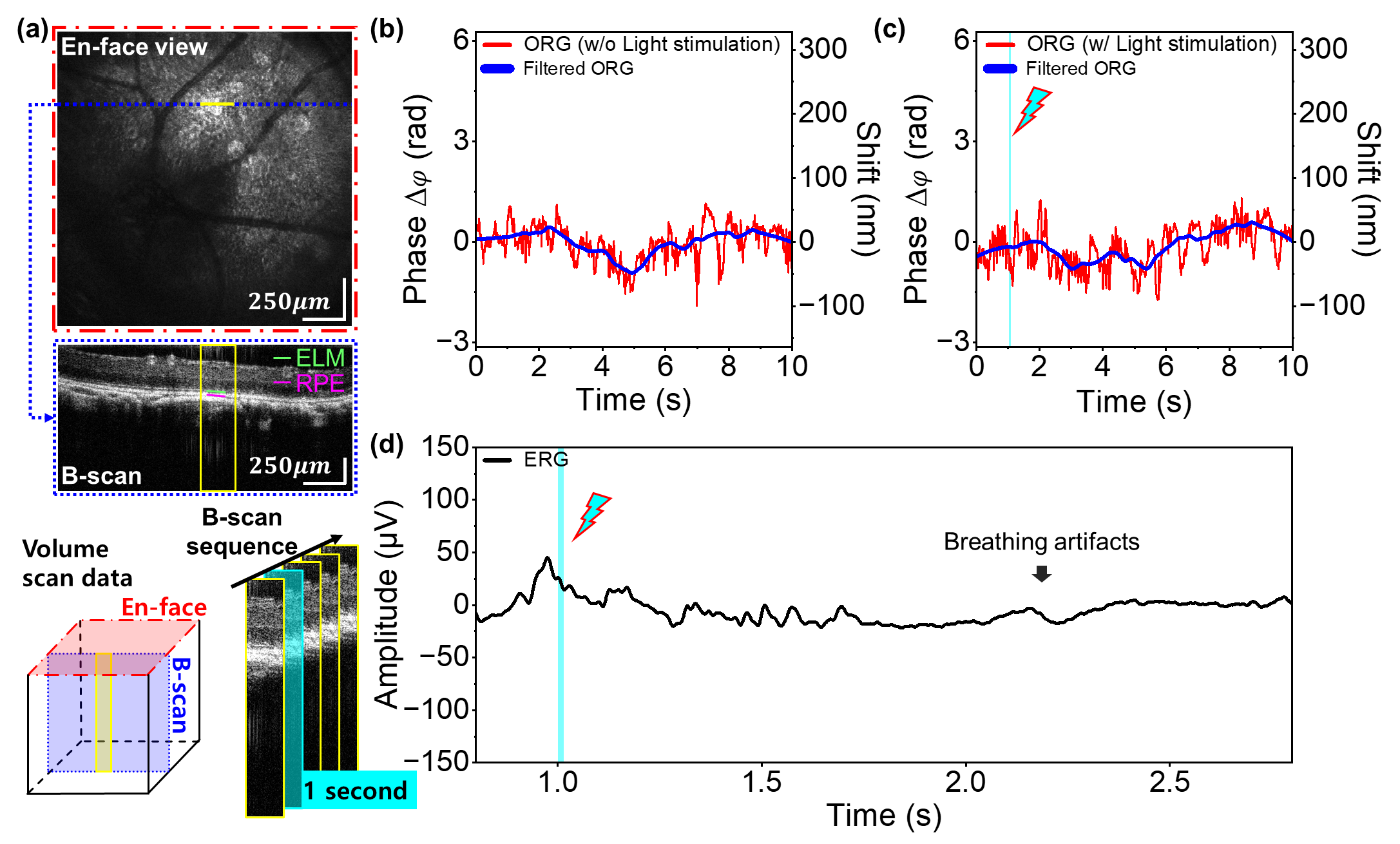


Fig. S12 Light-driven ORG and ERG signals with 47$.92 \mu J$ for 20% bleaching in pigmented retinal degeneration mice (rd10). (a) BM-scan area and distance measuring target (RPE to ELM). The parts marked in light blue in (a), (c), and (d) signified the light stimulation timing. (b) ORG signal for control without light stimulation. Since the rd10 models already had damage on the retinal layers, including the photoreceptor layers, the B-scan in (a) showed a shallower retina than those in Figs. 4(a) and Fig. 5(a). (c) ORG signal with light stimulation. The ORG signals in (b) and (c) showed minor changes, which are difficult to ascribe to light stimulation. To provide clear ORG signals, 0.05 – 60Hz bandpass filter and Savitzky-Golay filter were applied. (d) ERG graph showing only repeated wave responses that resembled breathing artifacts without any a-wave or b-wave.

# Section 11. Extended ERG signals


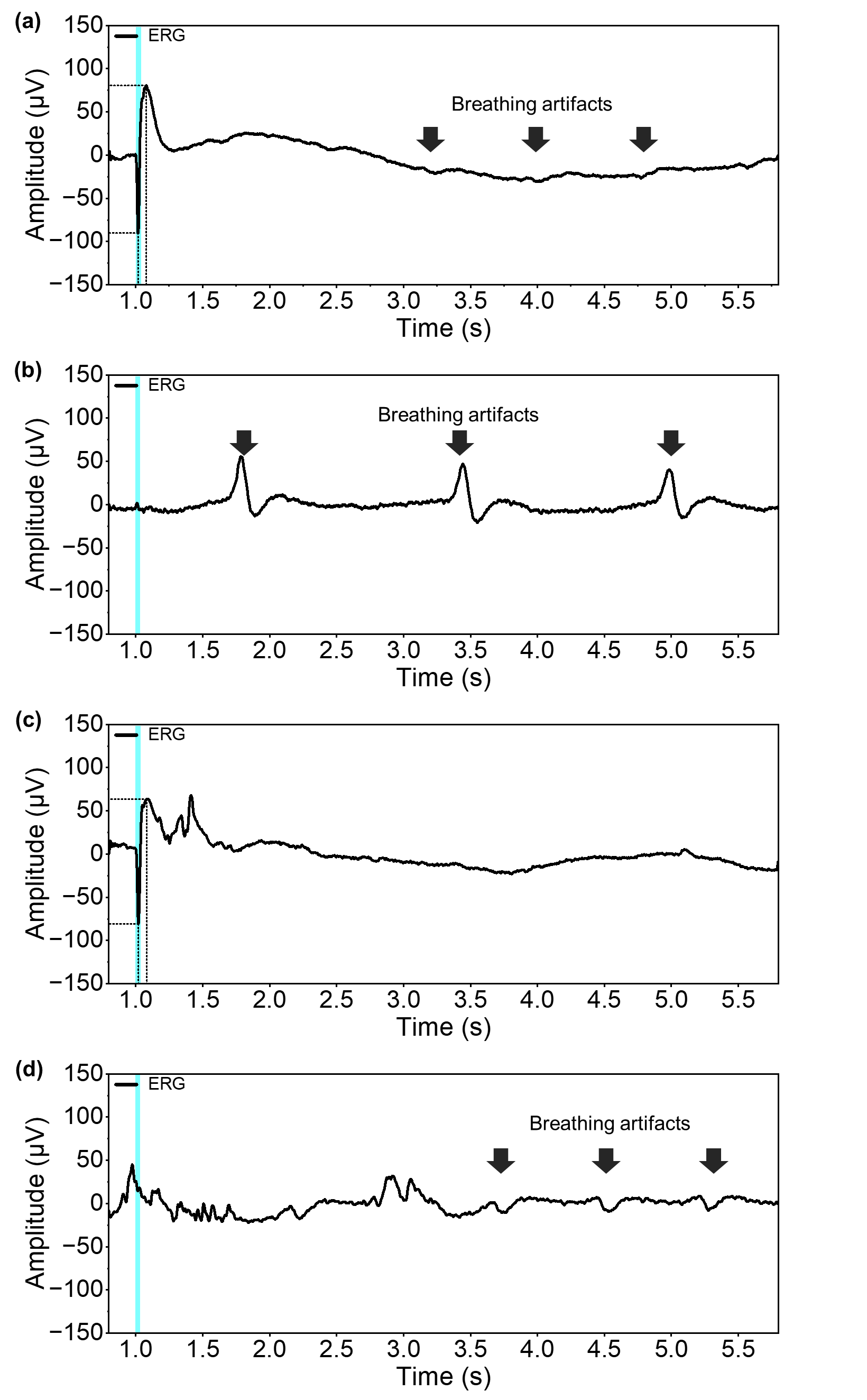


**Fig. S13** The extension of the each ERG signal. (a) Extended ERG of Fig. 5(d). (b) Extended ERG of Fig. 6(d). (c) Extended ERG of Fig. S10(d). (d) Extended ERG of Fig. S11(d).
